# Supplementary material for: Pathway centric analysis for single-cell RNA-seq and spatial transcriptomics data with GSDensity
Source: Nat Commun. 2023 Dec 18;14:8416. doi: 10.1038/s41467-023-44206-x (PMC10728201; doi:10.1038/s41467-023-44206-x)
Supplement: Supplementary file 1 — Supplementary Information [file 41467_2023_44206_MOESM1_ESM.pdf]

Supplementary Information

Pathway Centric Analysis for single-cell RNA-seq and Spatial Transcriptomics Data with GSDensity

Qingnan Liang[1], Yuefan Huang[1], Shan He[1], Ken Chen[1]

1. Department of Bioinformatics and Computational Biology, UT MD Anderson Cancer Center, Houston, Texas, United States

Supplementary Notes

Supplementary information of the TNBC data analysis.

With the TNBC dataset, we were particularly interested the question whether the tumor cells showed heterogeneity form the angle of their ability of proliferation. We used GSDensity to quantify the heterogeneity of the G2M checkpoint pathway among tumor cells and fetched the actively dividing tumor cells. To generally evaluate the heterogeneity of the G2M checkpoint genes among all 'hallmark' gene sets, we ran GSDensity for all 50 hallmark gene sets. The G2M checkpoint gene set was the 5th most heterogeneous according to the p-values. Moreover, we found that the subpopulation of interest also enriches other hallmark gene sets such as mitotic spindle or glycolysis (Fig 3b-d, p-value < 2.2e-16 for all three gene sets, Chi-squared test), which were all features of actively dividing cells, and thus enhanced our confidence in such observations (not being artifacts). Such enrichments were also observed in TNBC datasets from other patient samples (Extended Data Fig.6d-k).
We also assessed whether such a group of cells could be identified from a routine 'cluster-centric' approach. We found the actively dividing cells appeared as disjoint subpopulations in all four CNV-based clusters (Fig.3f). For transcriptome-based clustering, we clustered the datasets with different 'resolution' parameters to find if there were clusters that enrich the actively dividing tumor cells we identified. We tried to use Silhouette score as a metric to help find a good parameter of the 'resolution' (Fig.3g). With this metric, the best parameter would be 0.1 and result in 2 clusters. For all the resolution tested, under the best case, we found one cluster having only about half (54.5%) of the actively dividing tumor cells (resolution set at 1.2) identified by GSDensity. In conclusion, the actively dividing cells we identified with GSDensity could not be found with cluster-centric approaches. With this example, we demonstrated that GSDensity allowed for knowledge (pathways) guided analysis of limited single-cell data and can effectively generate novel, interpretable and testable hypotheses.

Supplementary Figures

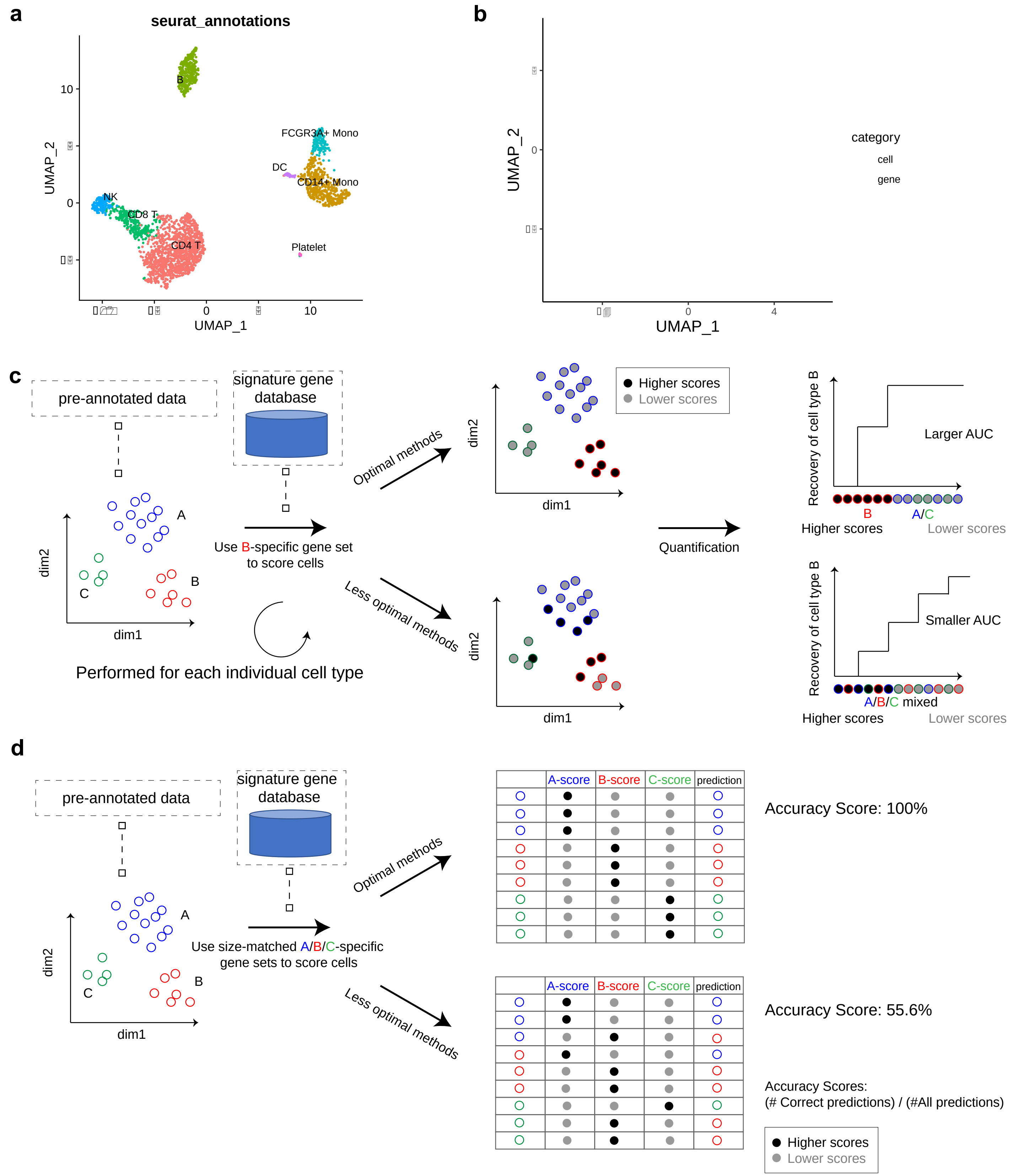

Supplementary Fig.1. Benchmarking gene set scoring algorithms using the pbmc3k scRNA-seq dataset.

a. UMAP visualization of cells in the pbmc3k dataset. Cells are colored based on their annotation. The UMAP embeddings were calculated based on gene expression.

b. UMAP visualization of MCA embedding of cells and genes of the pbmc3k dataset. Please notice that this UMAP took the MCA embeddings as the input, while all other UMAP embeddings were calculated using PCA dimensionality reductions for gene expressions.
c. Schematic of the benchmarking metric referred to as the AUC score. We use cell type signature genes curated from public database to score cells in pre-annotated scRNA-seq datasets. Optimal methods will have the matching cell types scored with high scores, which can be reflected with a larger AUC of the recovery curve.
d. Schematic of the benchmarking metric referred to as the ACC (accuracy) score. We use cell type signature genes curated from public database to score cells in pre-annotated scRNA-seq datasets. Thus, for each cell, it has a gene set score for each marker list, and it will be predicted to the cell type whose marker gets the highest score. The overall performance of methods are evaluated by the overall prediction accuracy.

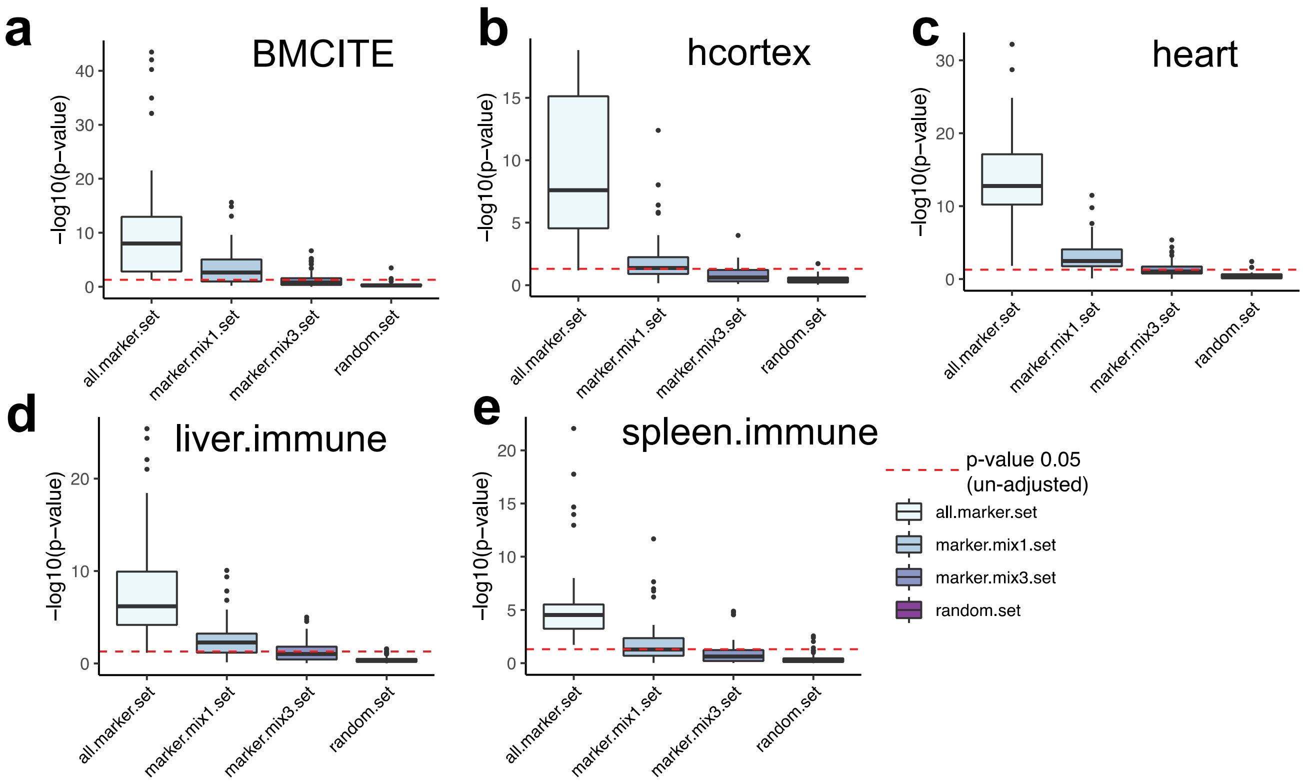

Supplementary Fig.2. Validation of the sensitivity of GSDensity to identify gene sets with coordination, related to Figure 2. Cell type markers (‘all.marker.set’), markers with size-matched random genes (‘marker.mix1.set’), markers with three-folds size-matched random genes (‘marker.mix3.set’), and all random genes (‘random.set’), were used as input gene sets to calculate their coordination in the ‘BMCITE’ (a), ‘hcortex’ (b), ‘heart’ (c), ‘liver.immune’ (d), and ‘spleen.immune’ (e) data, respectively. The red dashed line showed the unadjusted p-value equal to 0.05. One-sided t-test was used (Method). The center line of the box plot showed the median of data; the box limits showed the upper and lower quartiles; the whiskers showed 1.5 times interquartile range and points showed outliers. n = 40 for each box. Source data are provided as a Source Data file.

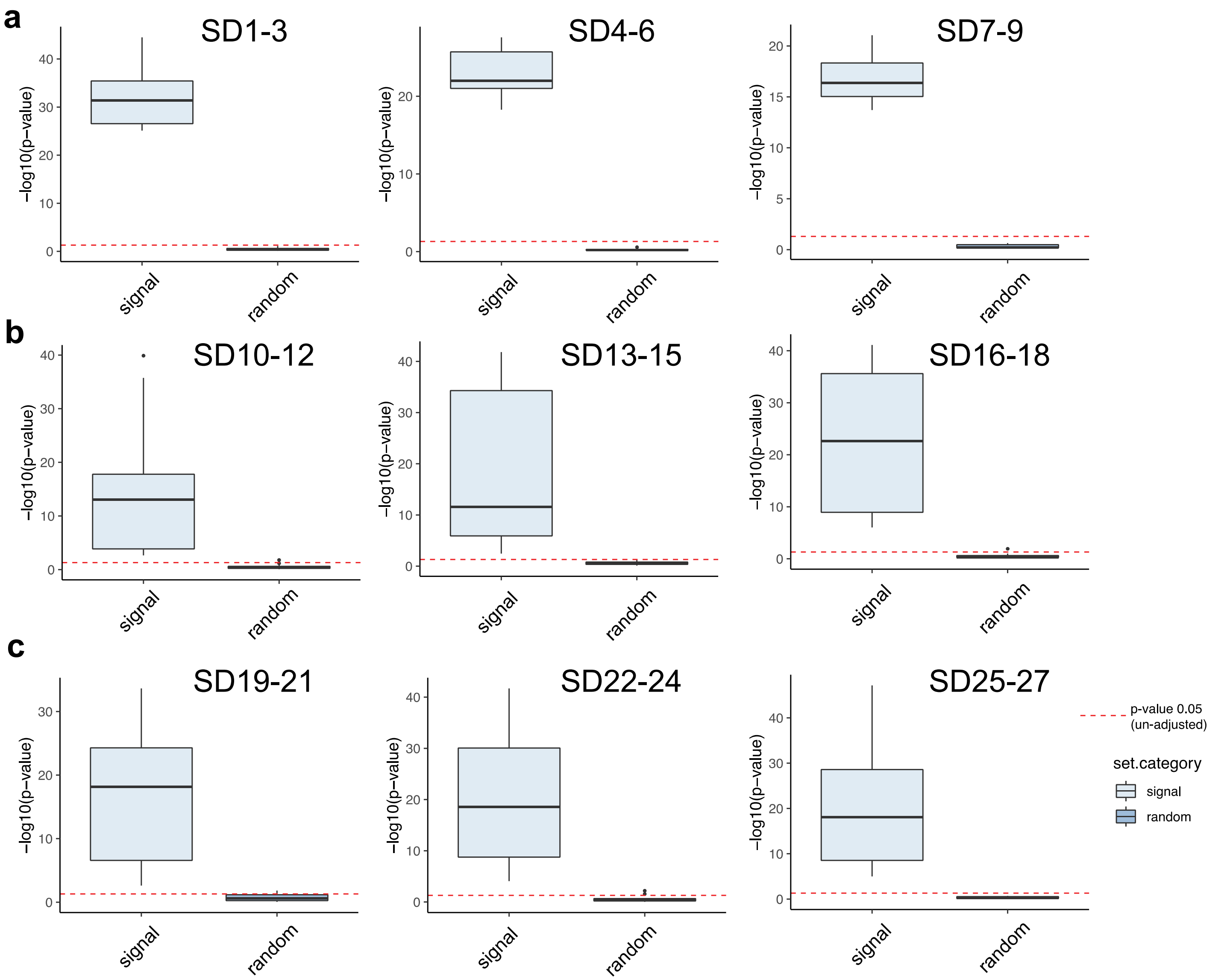

Supplementary Fig.3. Validation of the sensitivity of GSDensity to identify gene sets with coordination, using simulated data.

a-c. Validation experiments on simulation data of Mode-1 (a), Mode-2 (b), and Mode-3 (c). Ground-truth marker genes and randomly constructed gene sets were used as inputs for GSDensity on corresponding simulated datasets. The red dashed line showed the unadjusted p-value equal to 0.05. One-sided t-test was used (Method). The center line of the box plot showed the median of data; the box limits showed the upper and lower quartiles; the whiskers showed 1.5 times interquartile range and points showed outliers. n = 9 for each box. Source data are provided as a Source Data file.

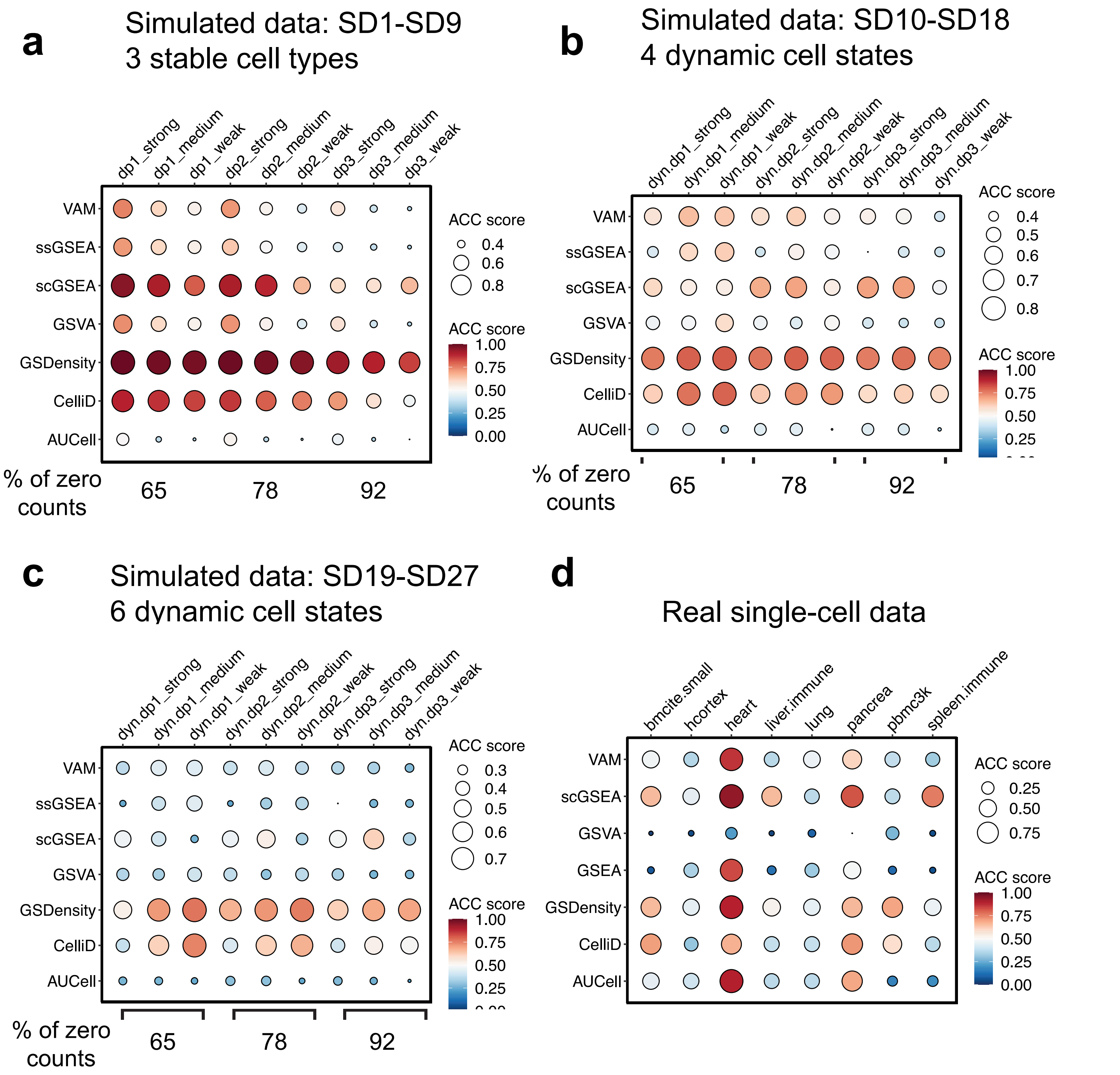

Supplementary Fig.4. Using the ACC score to evaluate the reliability of gene set scoring of GSDensity and six popular tools.

a-d. Benchmarking experiments on simulated datasets (a-c) and real-world datasets (d). Each row represents a method, and each column represents the gene set and dataset condition. For simulated datasets, the columns represent the average of three parallel datasets of the same mode and noise level. The colors and the sizes of the dots both demonstrate the ACC score. The colors and the sizes of the dots both demonstrate the ACC score. Source data are provided as a Source Data file.

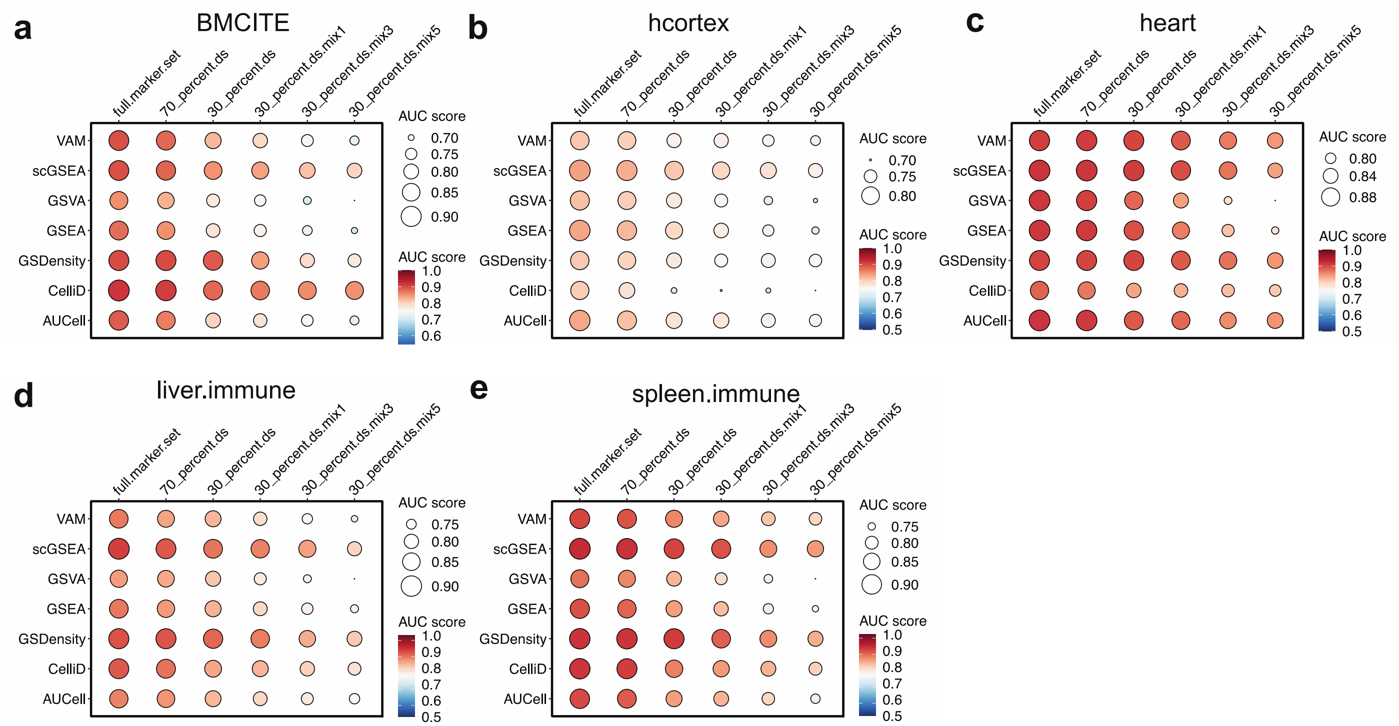

Supplementary Fig.5. Benchmarking the reliability of gene set scoring of GSDensity and six popular tools on real-world datasets.

a-e. Benchmarking experiments using the AUC score. Each row represents a method, and each column represents the gene set condition. The colors and the sizes of the dots both demonstrate the AUC score. The colors and the sizes of the dots both demonstrate the AUC score. Source data are provided as a Source Data file.

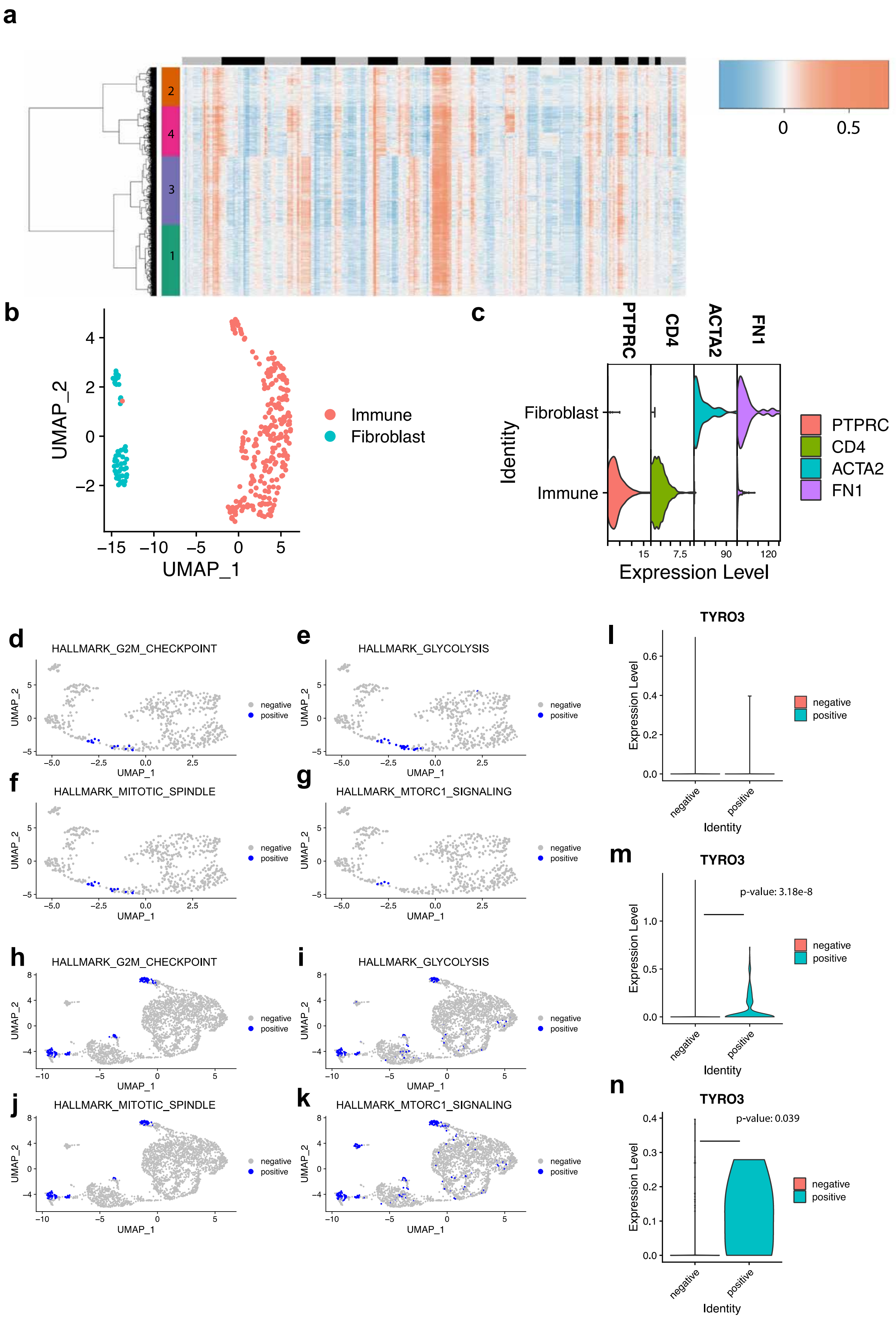
a
0
0.5
1
2
3
4
b
UMAP_2
UMAP_1
Immune
Fibroblast
c
PTPRC
CD4
ACTA2
FN1
Identity
Fibroblast
Immune
Expression Level
d
HALLMARK_G2M_CHECKPOINT
e
HALLMARK_GLYCOLYSIS
f
HALLMARK_MITOTIC_SPINDLE
g
HALLMARK_MTORC1_SIGNALING
h
HALLMARK_G2M_CHECKPOINT
i
HALLMARK_GLYCOLYSIS
j
HALLMARK_MITOTIC_SPINDLE
k
HALLMARK_MTORC1_SIGNALING
negative
positive
l
TYRO3
m
TYRO3
p-value: 3.18e-8
n
TYRO3
p-value: 0.039
Expression Level
Identity

Supplementary Fig. 6: GAS6-TYRO3 axis in dividing and non-dividing tumor cells in TNBC datasets.

a. Heatmap visualization of the TNBC-1 scRNA-seq dataset. Each row is a cell, and each column is a gnomic region. Warmer colors represent higher copy-numbers of that region. The cells (rows) are grouped with hierarchical clustering.

b. UMAP visualization of immune cells and fibroblasts in the TNBC-1 dataset. The UMAP embeddings were calculated based on gene expression.

c. Demonstration of immune and fibroblast markers (immune: CD4, PTPRC; fibroblast: ACTA2, FN1) in immune cells and fibroblast cells.

d-g. UMAP visualization of the TNBC-2 data using relevance to hallmark genes to classify cells. Cells which are the most relevant to the hallmark are labeled as ‘positive’. The UMAP embeddings were calculated based on gene expression.

h-k. UMAP visualization of the TNBC-5 data using relevance to hallmark genes to classify cells. Cells which are the most relevant to the hallmark are labeled as ‘positive’. The UMAP embeddings were calculated based on gene expression.

l-m. The expression of TYRO3 in G2M checkpoint positive and negative tumor cells in TNBC-2 (l), and TNBC5 (m) datasets. Wilcoxon test (two-sided) was applied here.

n. The expression of TYRO3 in G2M checkpoint positive and negative tumor cells in the GSM4909284_TN-MH0114-T2 dataset.

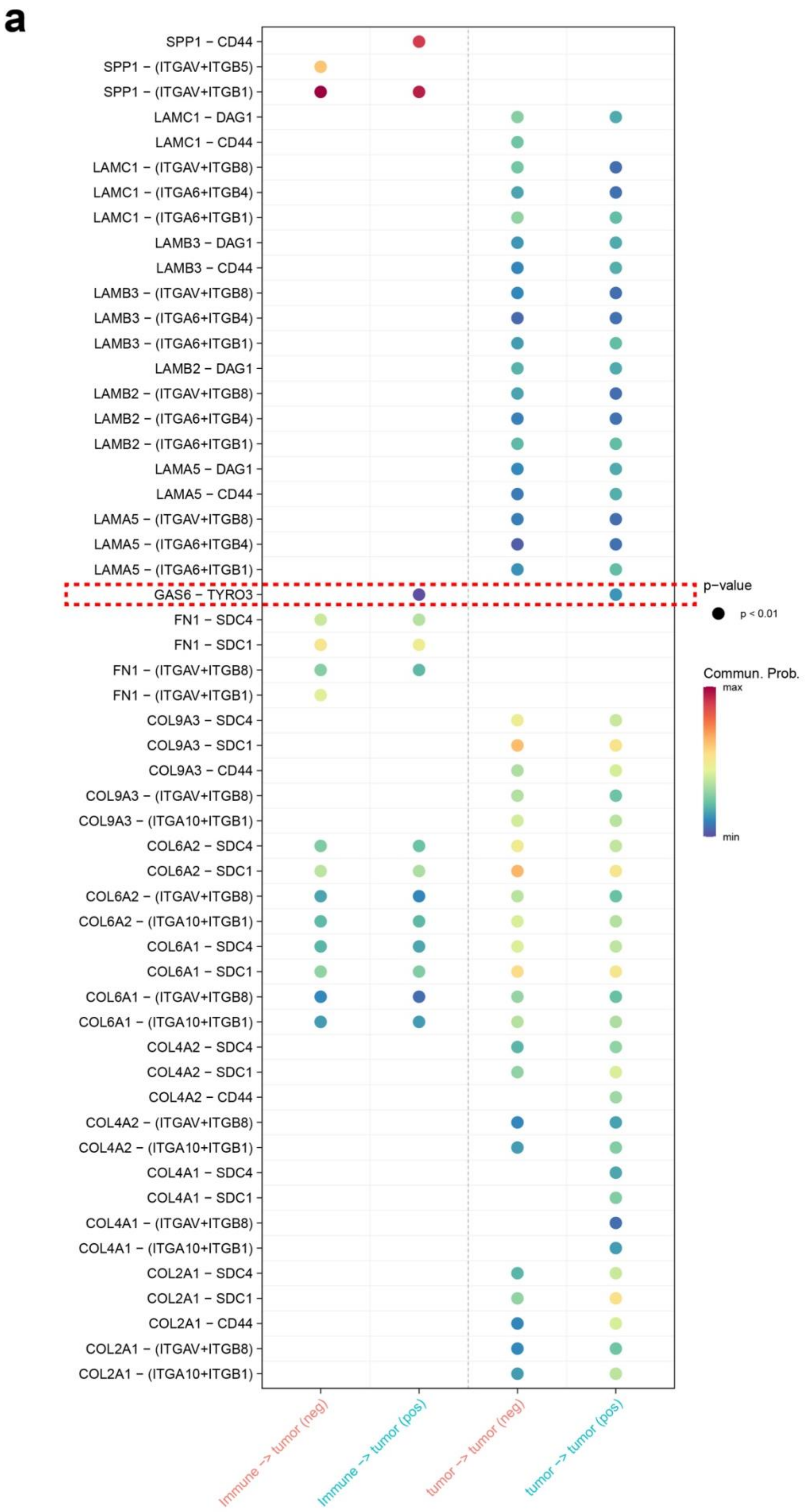
a
SPP1 − CD44
SPP1 − (ITGAV+ITGB5)
SPP1 − (ITGAV+ITGB1)
LAMC1 − DAG1
LAMC1 − CD44
LAMC1 − (ITGAV+ITGB8)
LAMC1 − (ITGA6+ITGB4)
LAMC1 − (ITGA6+ITGB1)
LAMB3 − DAG1
LAMB3 − CD44
LAMB3 − (ITGAV+ITGB8)
LAMB3 − (ITGA6+ITGB4)
LAMB3 − (ITGA6+ITGB1)
LAMB2 − DAG1
LAMB2 − (ITGAV+ITGB8)
LAMB2 − (ITGA6+ITGB4)
LAMB2 − (ITGA6+ITGB1)
LAMA5 − DAG1
LAMA5 − CD44
LAMA5 − (ITGAV+ITGB8)
LAMA5 − (ITGA6+ITGB4)
LAMA5 − (ITGA6+ITGB1)
GAS6 − TYRO3
FN1 − SDC4
FN1 − SDC1
FN1 − (ITGAV+ITGB8)
FN1 − (ITGAV+ITGB1)
COL9A3 − SDC4
COL9A3 − SDC1
COL9A3 − CD44
COL9A3 − (ITGAV+ITGB8)
COL9A3 − (ITGA10+ITGB1)
COL6A2 − SDC4
COL6A2 − SDC1
COL6A2 − (ITGAV+ITGB8)
COL6A2 − (ITGA10+ITGB1)
COL6A1 − SDC4
COL6A1 − SDC1
COL6A1 − (ITGAV+ITGB8)
COL6A1 − (ITGA10+ITGB1)
COL4A2 − SDC4
COL4A2 − SDC1
COL4A2 − CD44
COL4A2 − (ITGAV+ITGB8)
COL4A2 − (ITGA10+ITGB1)
COL4A1 − SDC4
COL4A1 − SDC1
COL4A1 − (ITGAV+ITGB8)
COL4A1 − (ITGA10+ITGB1)
COL2A1 − SDC4
COL2A1 − SDC1
COL2A1 − CD44
COL2A1 − (ITGAV+ITGB8)
COL2A1 − (ITGA10+ITGB1)
Immune -> tumor (neg)
Immune -> tumor (pos)
tumor -> tumor (neg)
tumor -> tumor (pos)
p−value
p < 0.01
Commun. Prob.
max
min

Supplementary Fig.7: Ligand-receptor interactions between immune-tumor and tumor-tumor in TNBC-1 data. Tumor cells are classified based on their relevance to G2M checkpoint. Here only the ligand-receptor pairs showing differential communication level are plotted.

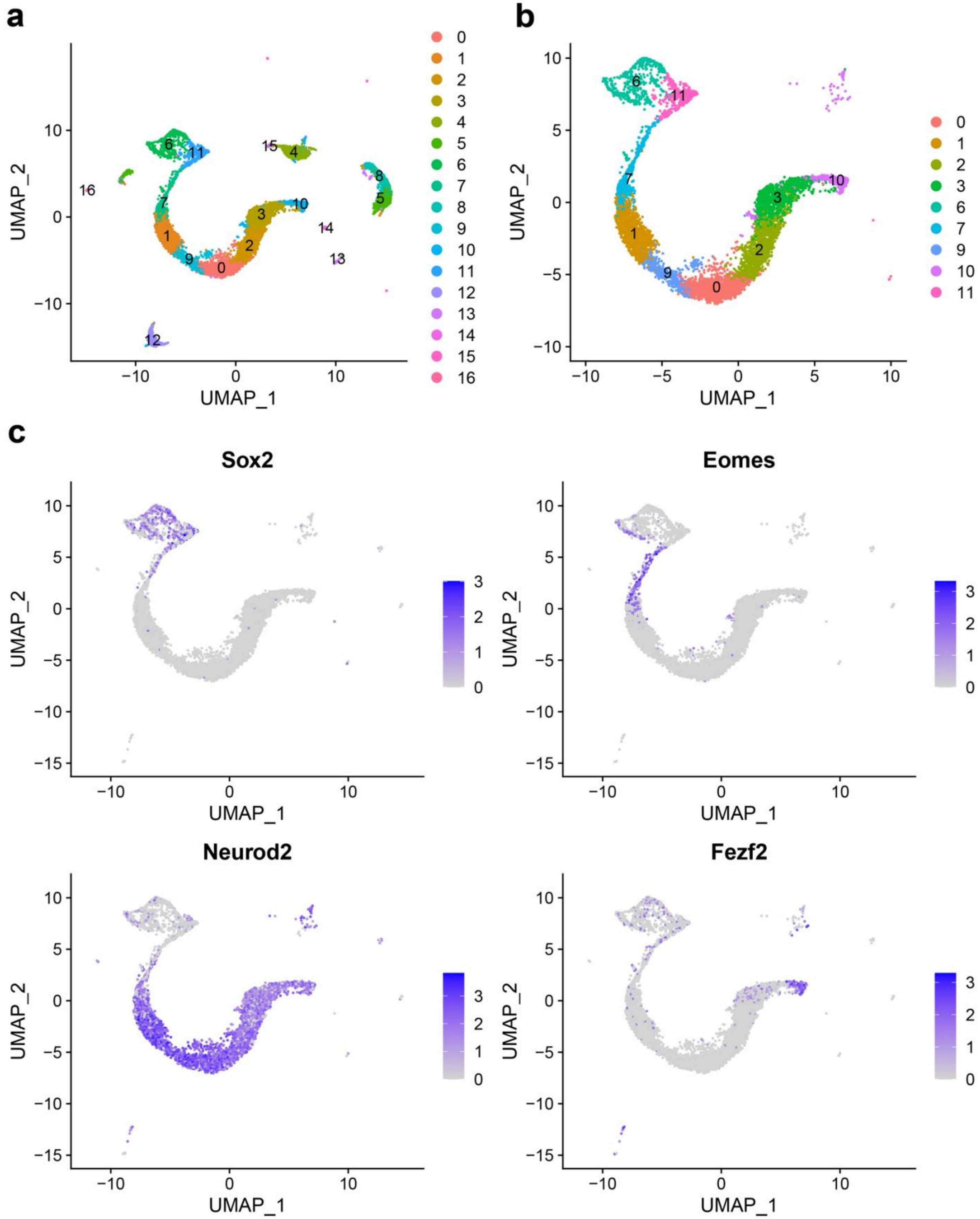

Supplementary Fig. 8: Determination of developmental stage of the E17.5 mouse cerebral cortex scRNA-seq data.

a. UMAP visualization of the E17.5 mouse cerebral cortex dataset. The cells are colored with the cluster names. The UMAP embeddings were calculated based on gene expression.

b. UMAP visualization of the clusters used for the following trajectory analysis.

c. Expression of marker genes for developmental stages in the datasets. This is for the purpose of determining the order of the trajectory.

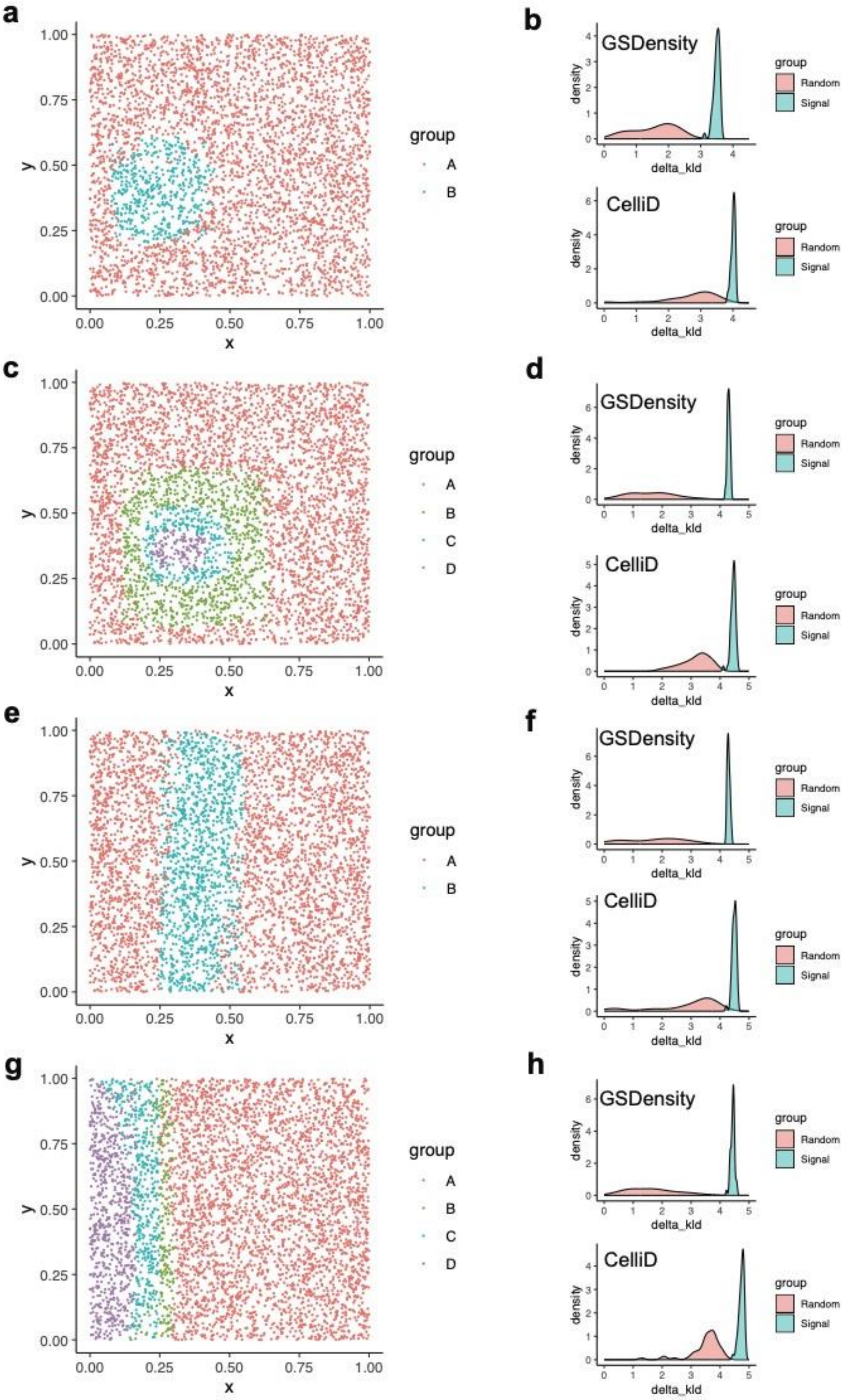

a
b
GSDensity
CelliD
c
d
e
f
g
h
x
y
group
A
B
C
D
density
delta_kld
Random
Signal

Supplementary Fig.9. Validating the performance of GSDensity in detecting spatially relevant gene sets.

a. Spatial organization of simulated clusters A and B in the ‘hotspot’ mode. Cluster B had higher expression levels of known signal genes in prior.

b. Distribution of the metric delta-KLD for signal gene sets and random gene sets using GSDensity (top) and CelliD (bottom) for PAL scoring for the hotspot mode data.

c. Spatial organization of simulated clusters A-D in the ‘hotspot with gradient’ mode. Cluster D had the highest expression levels of known signal genes in prior.

d. Distribution of the metric delta-KLD for signal gene sets and random gene sets using GSDensity (top) and CelliD (bottom) for PAL scoring for the hotspot with gradient mode data.

e. Spatial organization of simulated clusters A and B in the ‘streak’ mode. Cluster B had higher expression levels of known signal genes in prior.

f. Distribution of the metric delta-KLD for signal gene sets and random gene sets using GSDensity (top) and CelliD (bottom) for PAL scoring for the streak mode data.

g. Spatial organization of simulated clusters A-D in the ‘gradient’ mode. Cluster D had the highest expression levels of known signal genes in prior.

h. Distribution of the metric delta-KLD for signal gene sets and random gene sets using GSDensity (top) and CelliD (bottom) for PAL scoring for the gradient mode data.

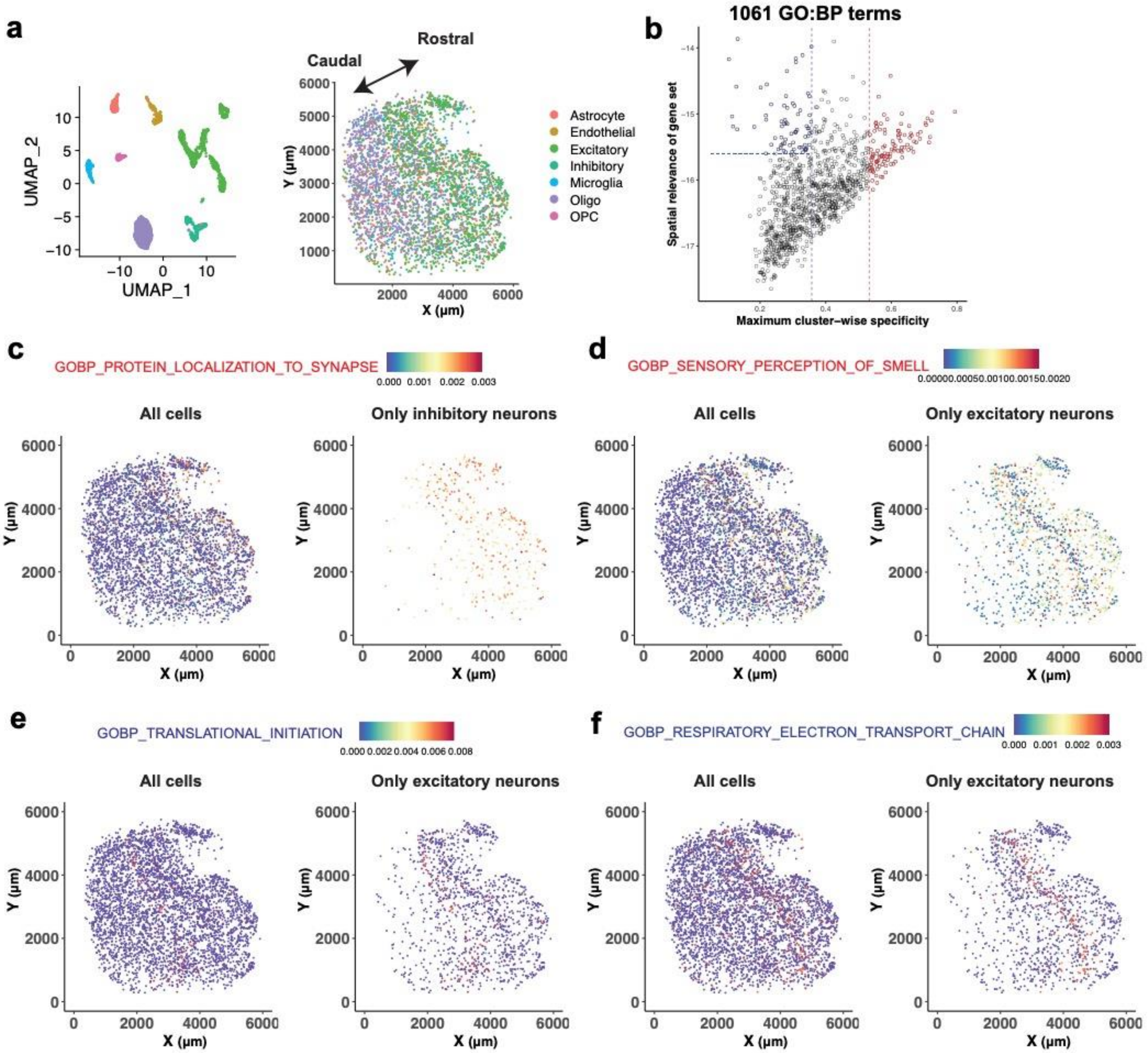

Extended Fig.10. Application of GSDensity to human prefrontal cortex data generated by Slide-tags.
a. The UMAP representation (left) and spatial localizations (right) of cells colored by the annotations. The UMAP embeddings were calculated based on gene expression.
b. Visualization of 1061 gene ontology (biological process) terms based on their cluster-wise specificity and spatial relevance. The gene ontology terms are the ones having significant coordination in the mouse anterior brain dataset. The data points highlighted with red represent the ones with high cluster-wise specificity. The data points highlighted with blue represent the ones with low cluster-wise specificity and high spatial relevance.
c-d. Example of pathways showing high cell-type specific PALs in inhibitory neurons (c) and excitatory neurons (d). For each panel, the cell type with high specificity to the pathway is plotted solely on the right to demonstrate the distribution of the PAL among this cell type.
e-f. Example of pathways showing low cell-type specific PALs in excitatory neurons. For each panel, the cell type with high specificity to the pathway is plotted solely on the right to demonstrate the distribution of the PAL among this cell type.

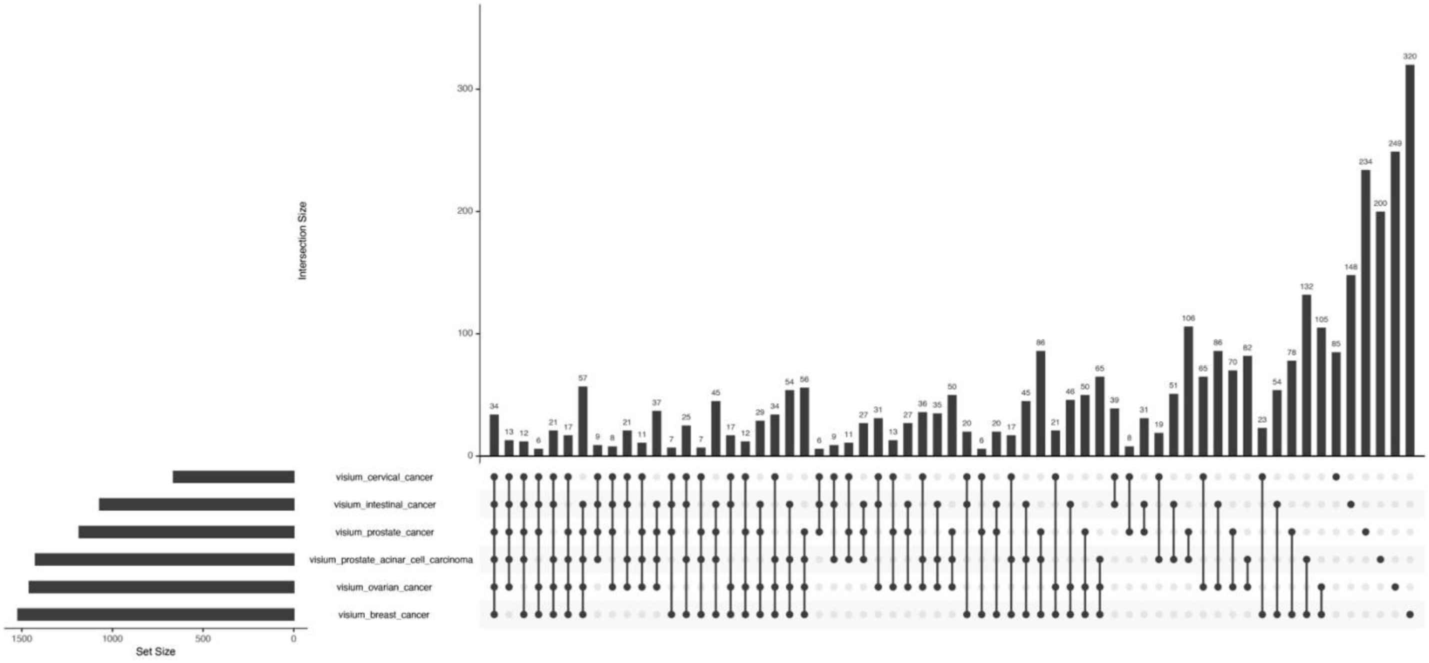

Supplementary Fig. 11. Spatially relevant pathways and their overlapping in six tumor spatial transcriptomics datasets.

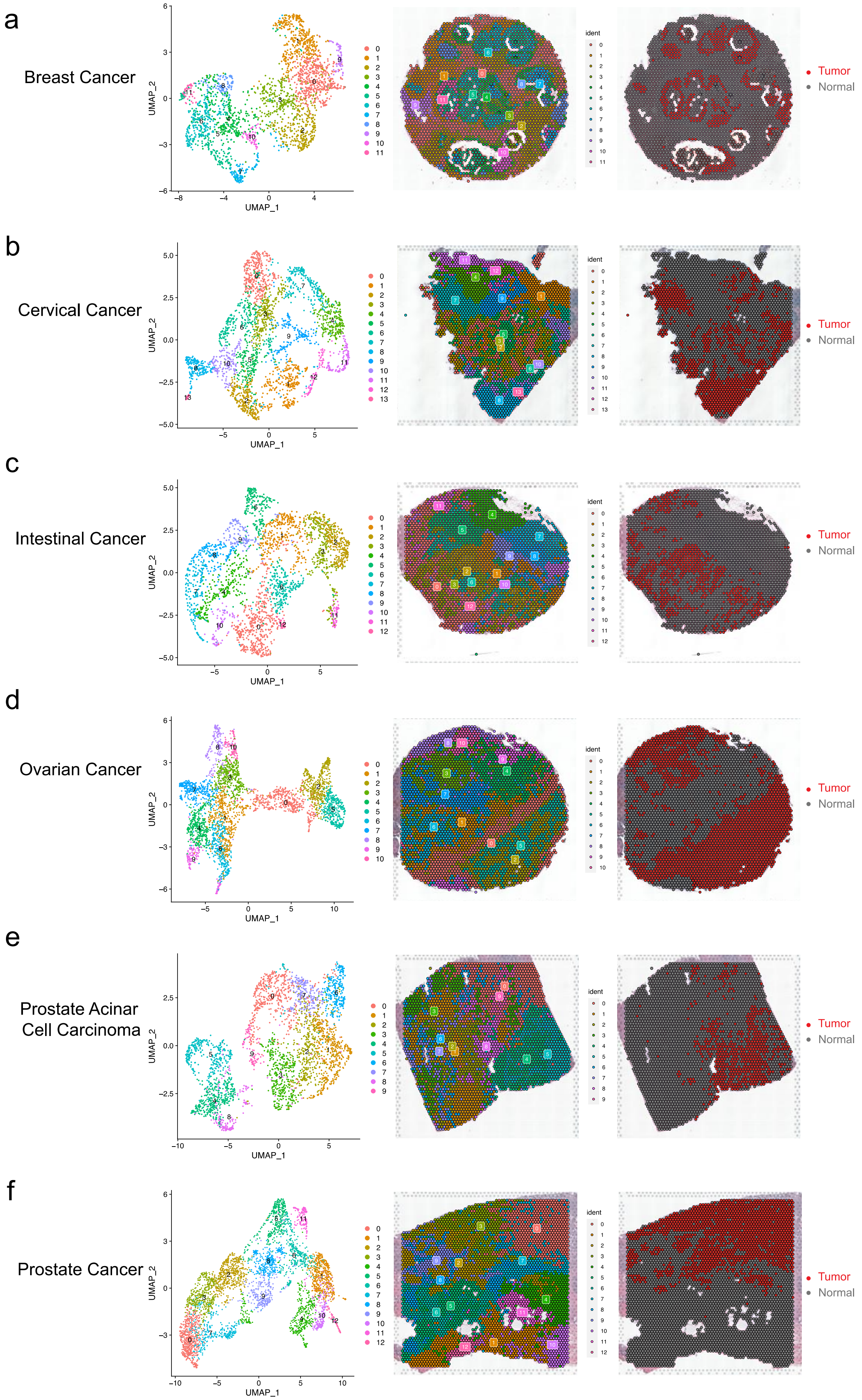
a
Breast Cancer
UMAP_1
UMAP_2
ident
Tumor
Normal
b
Cervical Cancer
UMAP_1
UMAP_2
ident
Tumor
Normal
c
Intestinal Cancer
UMAP_1
UMAP_2
ident
Tumor
Normal
d
Ovarian Cancer
UMAP_1
UMAP_2
ident
Tumor
Normal
e
Prostate Acinar
Cell Carcinoma
UMAP_1
UMAP_2
ident
Tumor
Normal
f
Prostate Cancer
UMAP_1
UMAP_2
ident
Tumor
Normal

Supplementary Fig. 12. Overview of the cancer spatial transcriptomics datasets.
a-f. Overview of breast cancer (a), cervical cancer (b), intestinal cancer (c), ovarian cancer (d), prostate acinar cell carcinoma (e), and prostate cancer (f). For each panel, UMAP visualization of cell clusters (left), mapping cell clusters to spatial maps (middle), and mapping predicted cancer cells to spatial maps (right) are visualized. The UMAP embeddings were calculated based on gene expression.

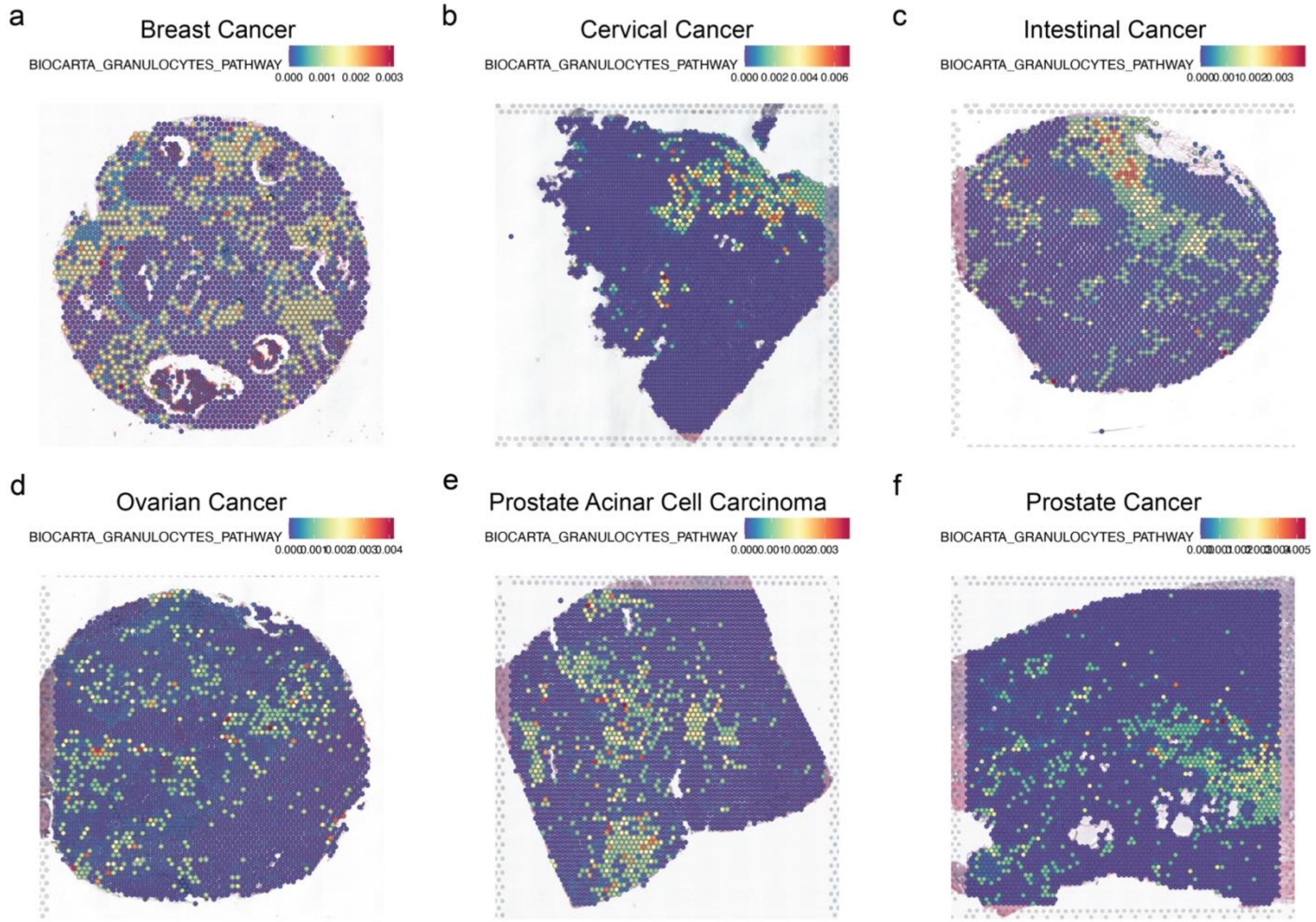

Supplementary Fig. 13. The spatial distribution of granulocyte pathway in tumor samples of different tumor types without highlighting tumor cells.

a-f. Visualization of granulocyte pathway in breast cancer (a), cervical cancer (b), intestinal cancer (c), ovarian cancer (d), prostate acinar cell carcinoma (e), and prostate cancer (f).

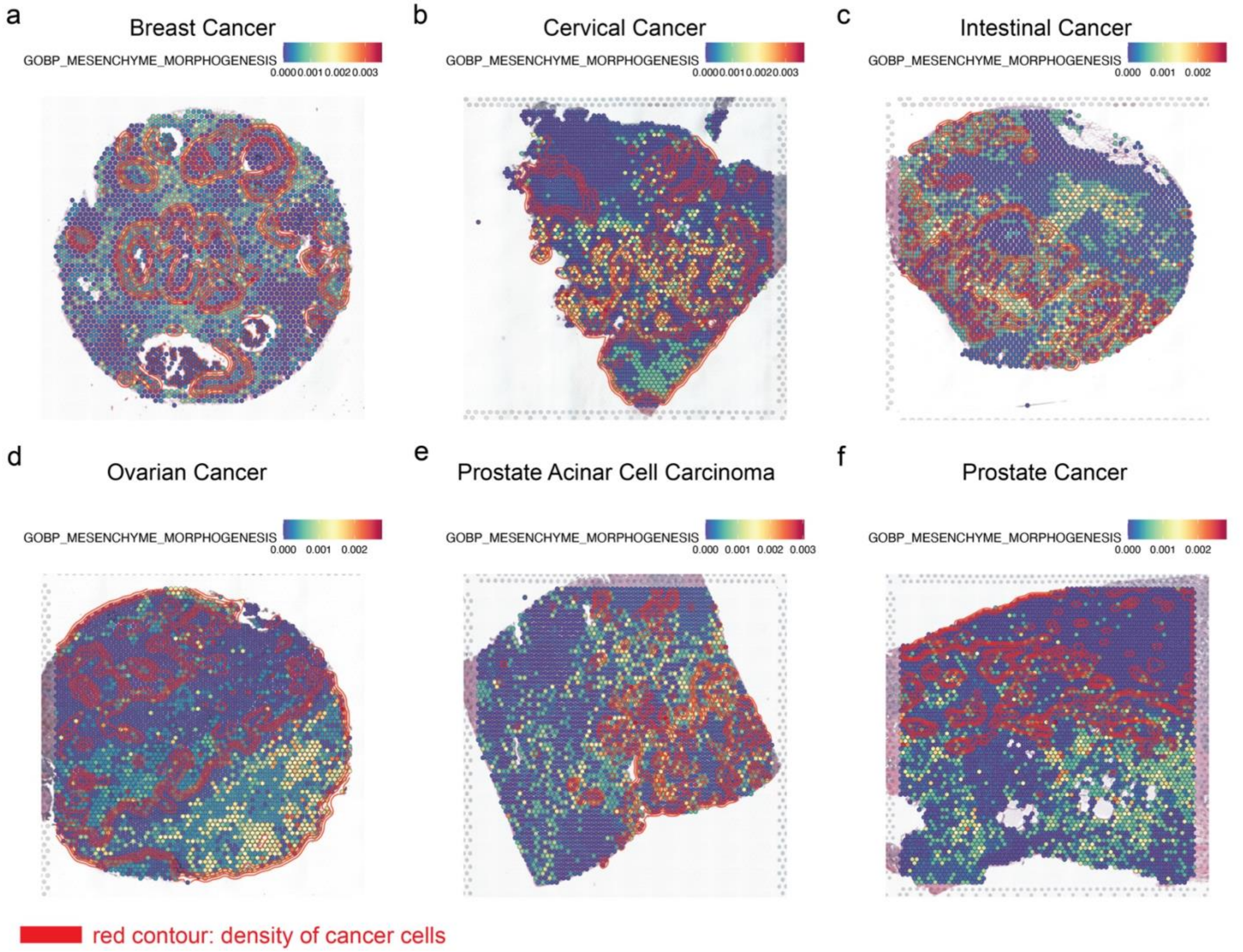

Supplementary Fig. 14: Applying GSDensity to reveal the spatial distribution of mesenchyme morphogenesis pathway in tumor samples of different tumor types.

a-f. Visualization of mesenchyme morphogenesis pathway in breast cancer (a), cervical cancer (b), intestinal cancer (c), ovarian cancer (d), prostate acinar cell carcinoma (e), and prostate cancer (f). Red contour lines represent the density of tumor cells.

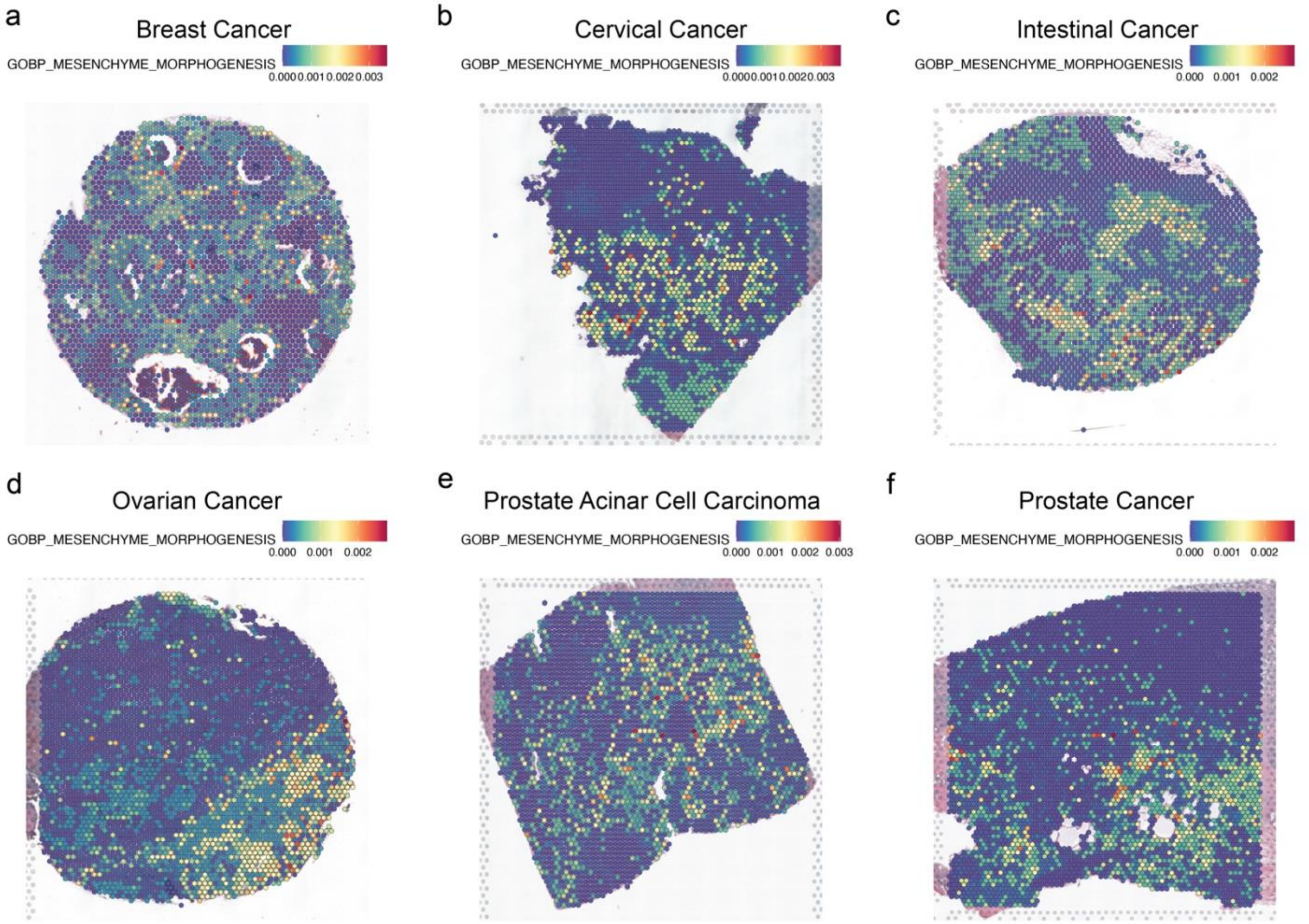

Supplementary Fig. 15. The spatial distribution of mesenchyme morphogenesis pathway in tumor samples of different tumor types without highlighting tumor cells.

a-f. Visualization of mesenchyme morphogenesis pathway in breast cancer (a), cervical cancer (b), intestinal cancer (c), ovarian cancer (d), prostate acinar cell carcinoma (e), and prostate cancer (f).

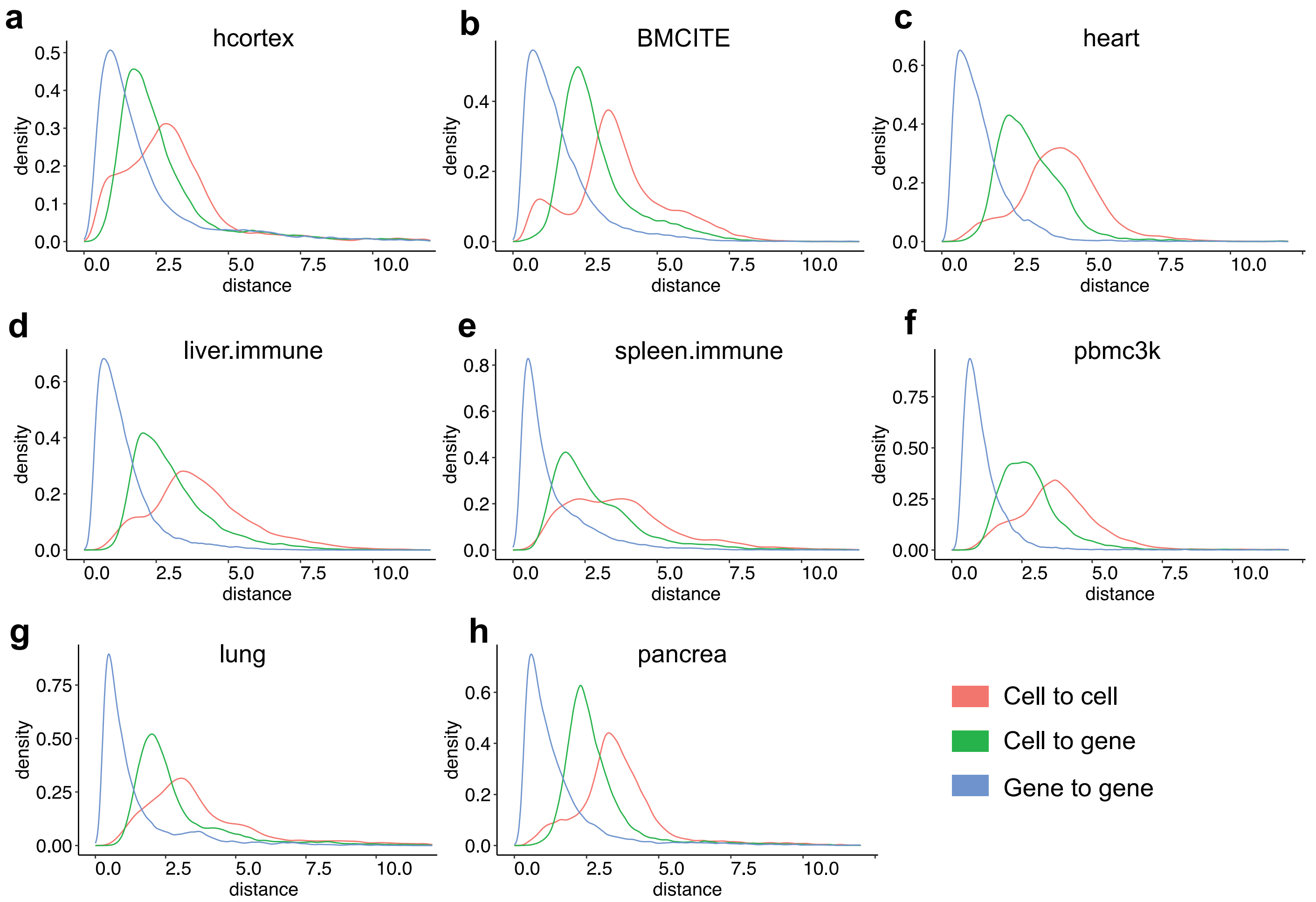

Supplementary Figure 16. Distribution of the distances of three categories (cell to cell, gene to gene, and cell to gene) in the MCA biplot.

a-h. Distance distributions for real-world datasets. For each dataset, each category of the distances was downsampled to 50,000 for the visualization. The distances were all Euclidean distances. Here we only showed the distances between 0 and 12, which contains the majority of the distances.

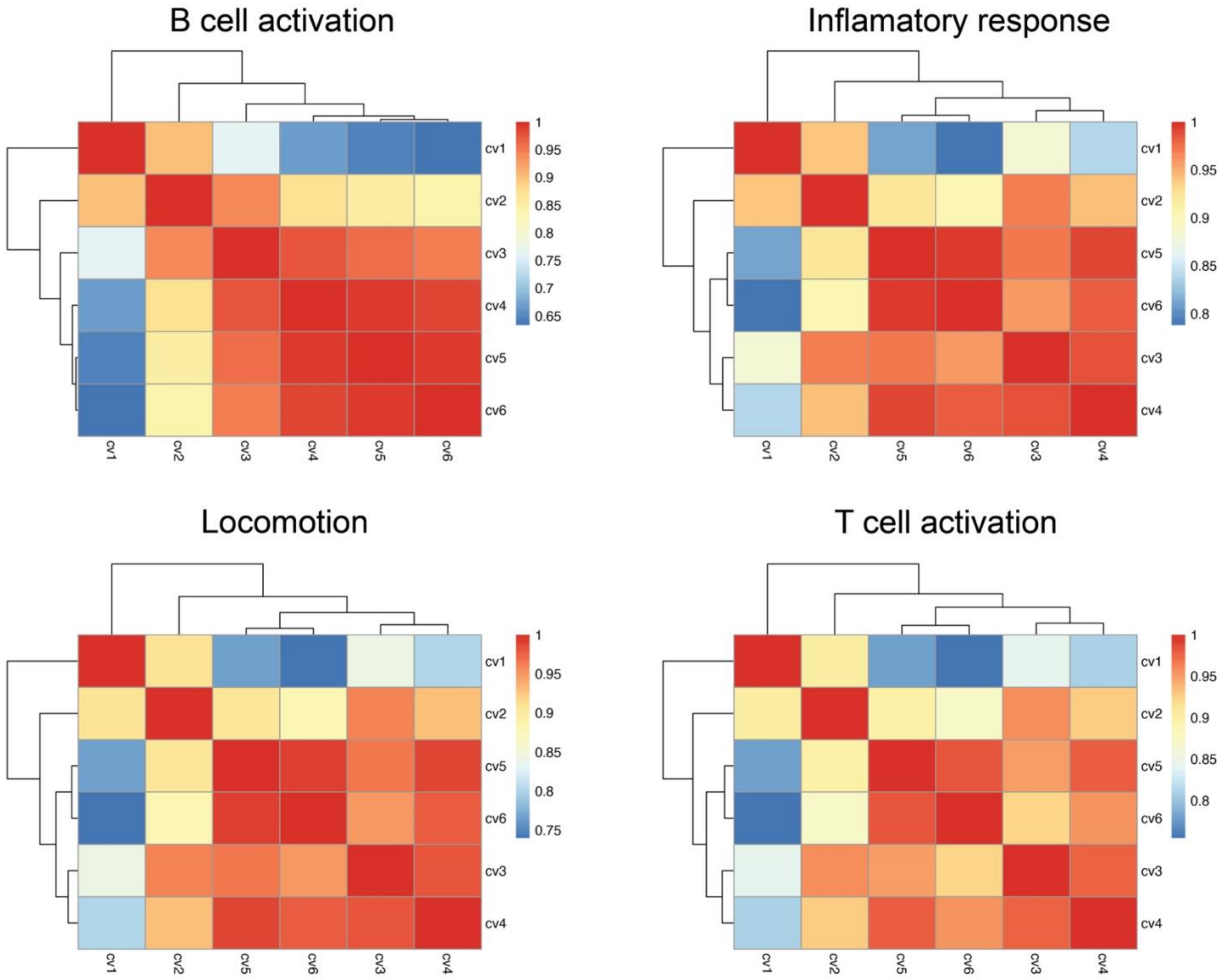

Supplementary Figure 17. The robustness of GSDensity PAL calculation to the parameter (number of neighbors) of the nearest neighbor graph used for network propagation. We show the correlation of single-cell PAL among six different parameters (number of neighbors: cv1: 100; cv2: 200; cv3: 300; cv4: 400; cv5: 500; cv6: 600) for four different pathways in the PBMC dataset.

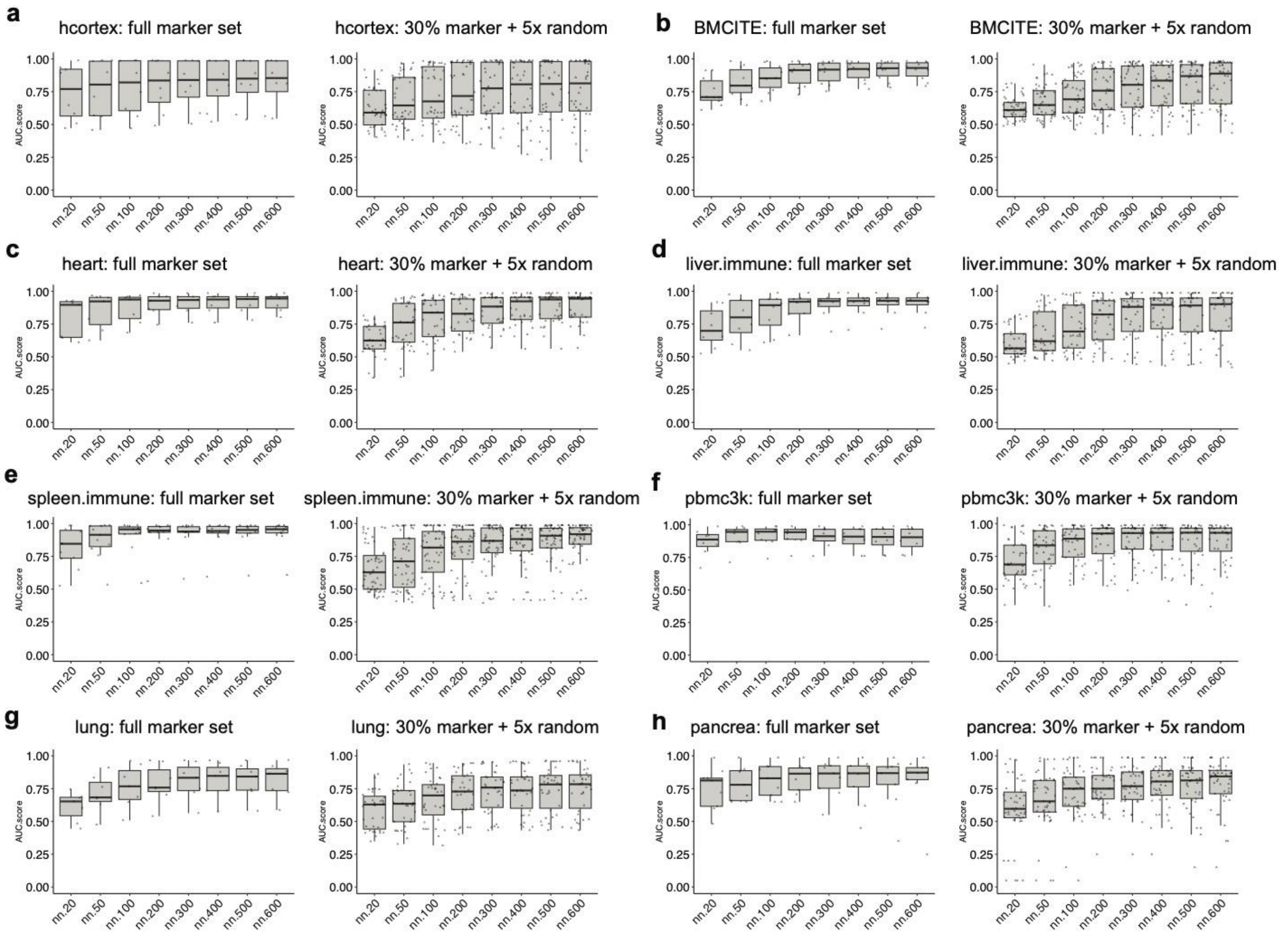

Supplementary Figure 18. The cell identity recovery using marker genes (AUC metric) with different numbers of nearest neighbors used in constructing the NN-graph in GSDensity.
a-h. AUC scores for each real-world dataset with different neighborhood sizes. For each panel, we demonstrated the recovery with full marker sets (left, strong specificity) or with marker sets mixed with random gene (right, weaker specificity). Sample sizes (n) equal to the number of cell types (left panels) and five times the number of cell types (right panels) as are listed in Table 1. Source data are provided as a Source Data file.

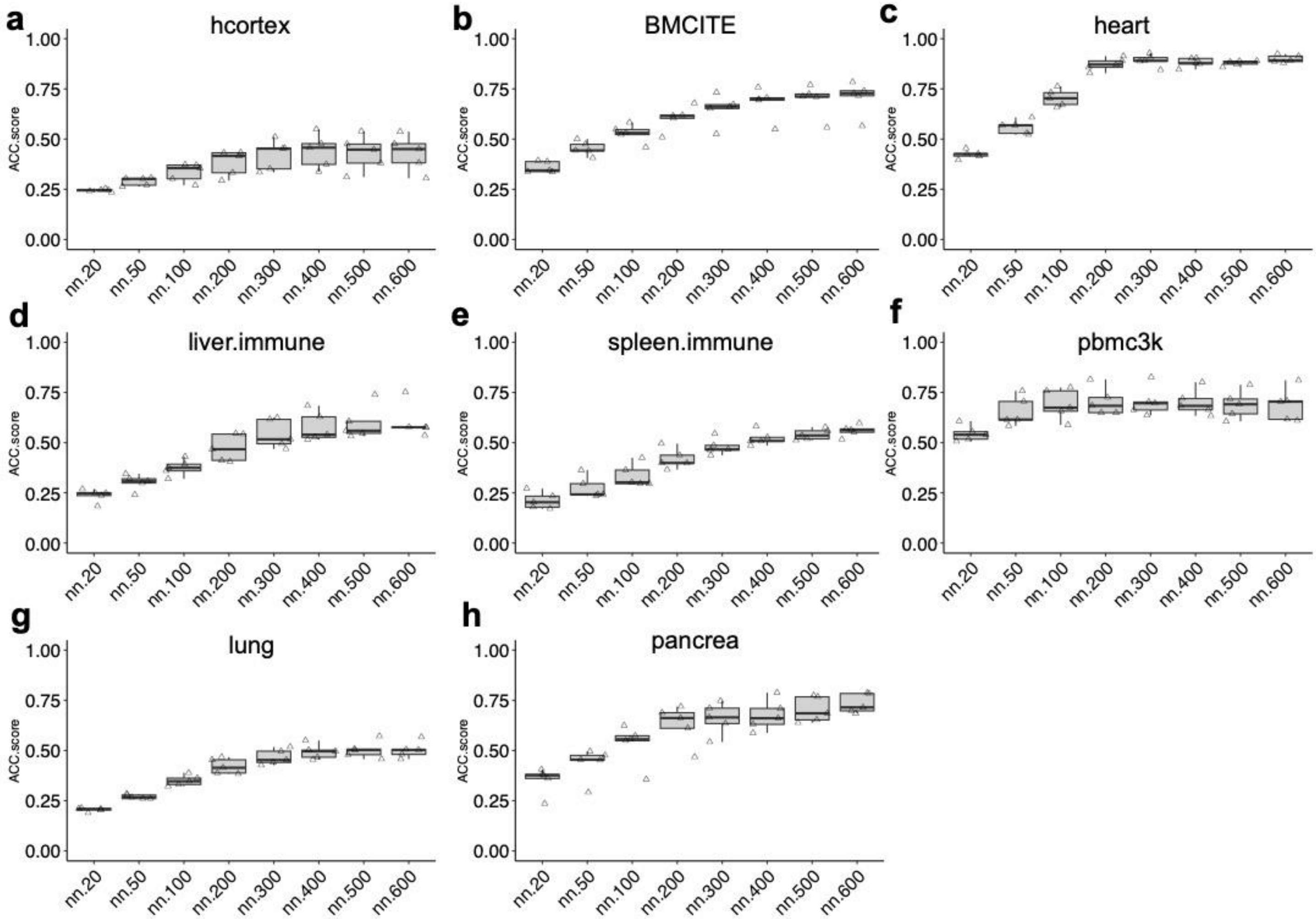

Supplementary Figure 19. The cell identity prediction using marker genes (ACC metric) with different numbers of nearest neighbors used in constructing the NN-graph in GSDensity.
a-h. ACC scores for each real-world dataset with different neighborhood sizes. Sample sizes (n) equal to the number of cell types as are listed in Table 1. Source data are provided as a Source Data file.

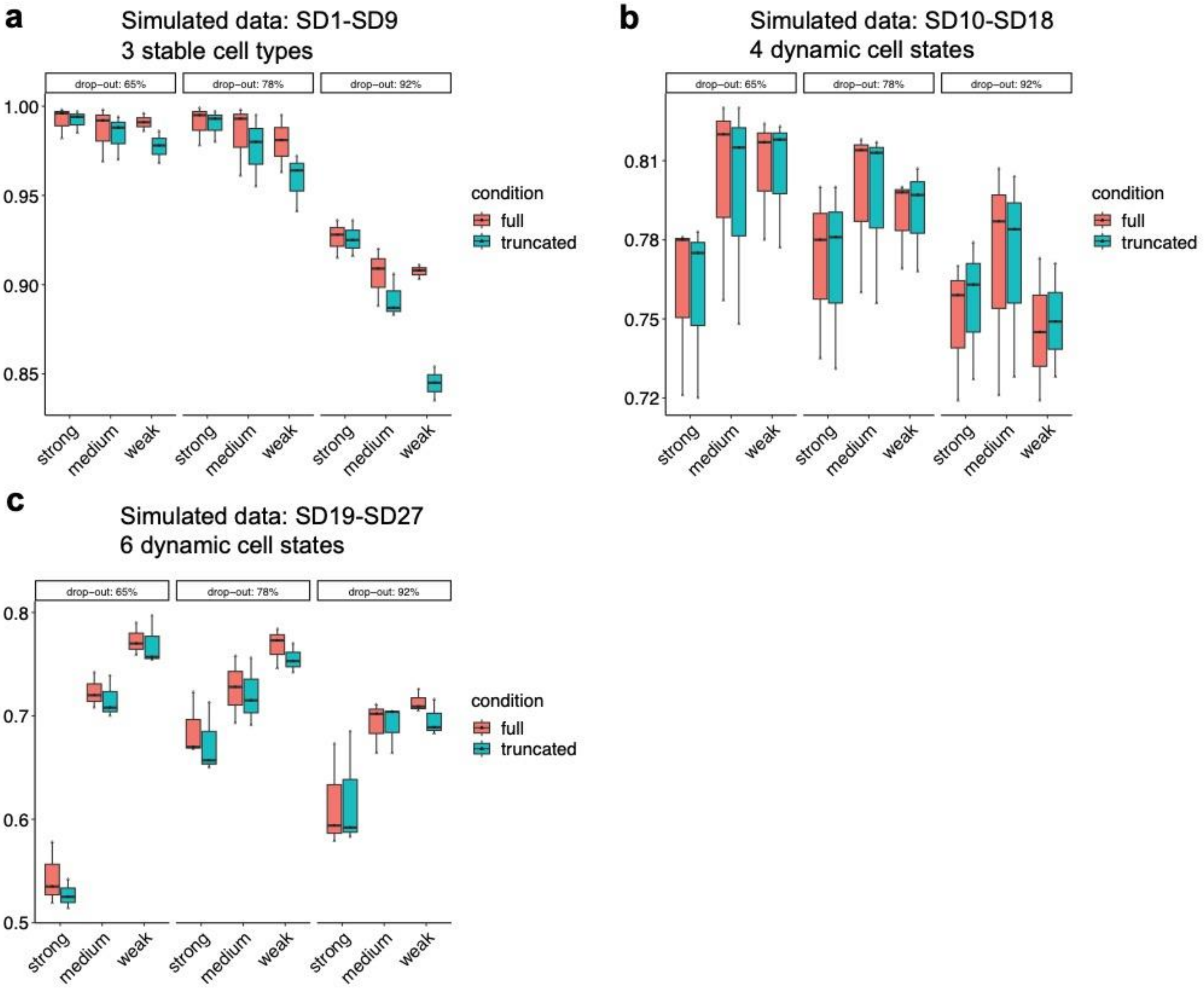

Supplementary Figure 20. GSDensity performance on cell identity prediction with truncated (to 10) marker sets and full marker sets (length varies) using simulated data (Mode-1, a; Mode-2, b; Mode-3, c). For each mode, the data with three different sparsity levels were investigated combined with marker sets with different signal strengths (strong, medium, and weak). n = 3 for each box. Source data are provided as a Source Data file.

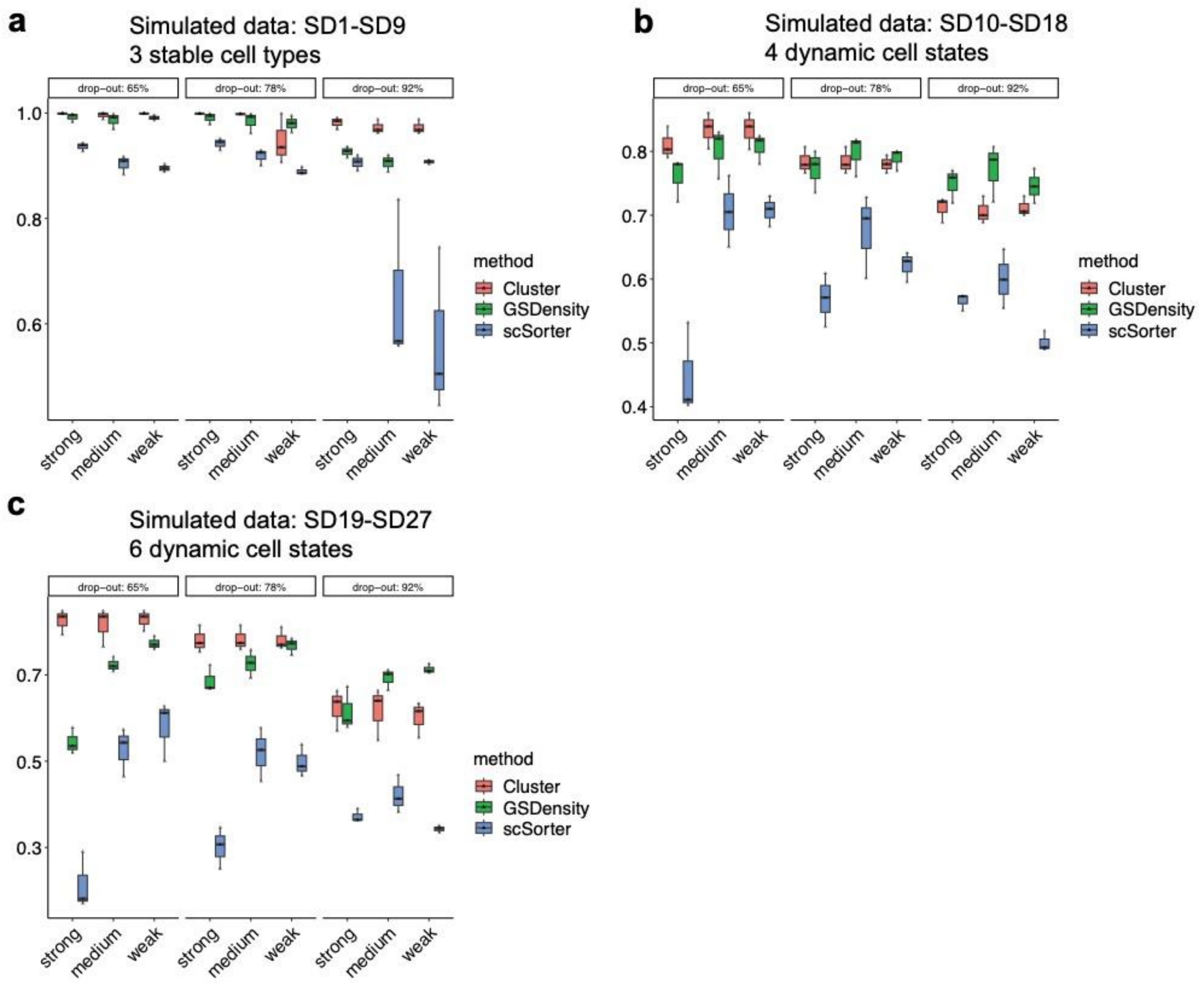

Supplementary Figure 21. Comparing the cell type annotation accuracy among GSDensity (green), scSorter (blue), and a cluster-based strategy (red, Methods) using simulated data (Mode-1, a; Mode-2, b; Mode-3, c). For each mode, the data with three different sparsity levels were investigated combined with marker sets with different signal strengths (strong, medium, and weak). For all three methods, full marker sets were used. n = 3 for each box. Source data are provided as a Source Data file.

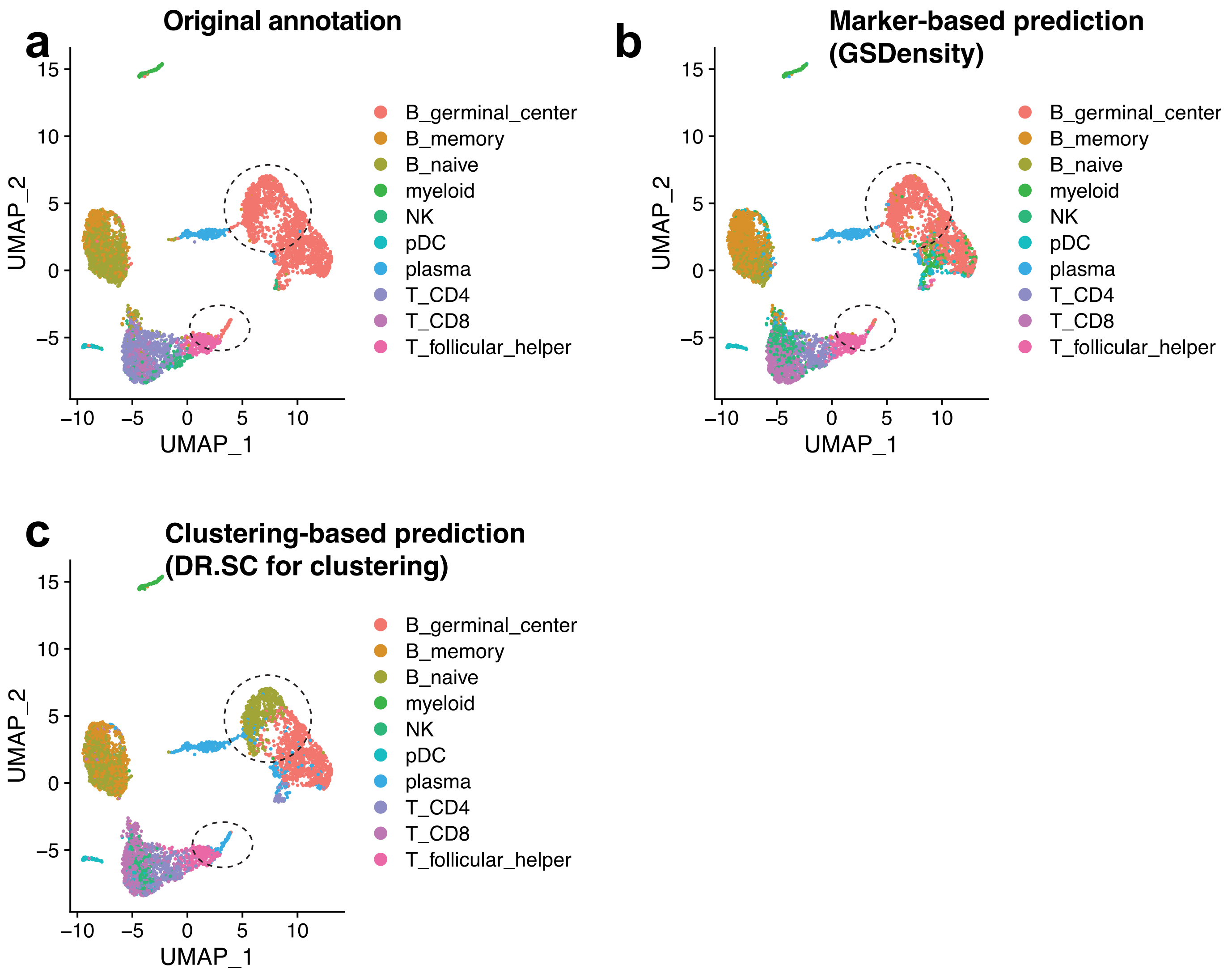

Supplementary Figure 22. Comparing the cell type annotation accuracy between GSDensity (and a cluster-based strategy (Methods) in a spatial genomics dataset. We demonstrate the cell labels from original annotation (a), GSDensity-based prediction (b) and clustering-based prediction (c). Prediction of cells with GSDensity showing significant improvement were highlighted.

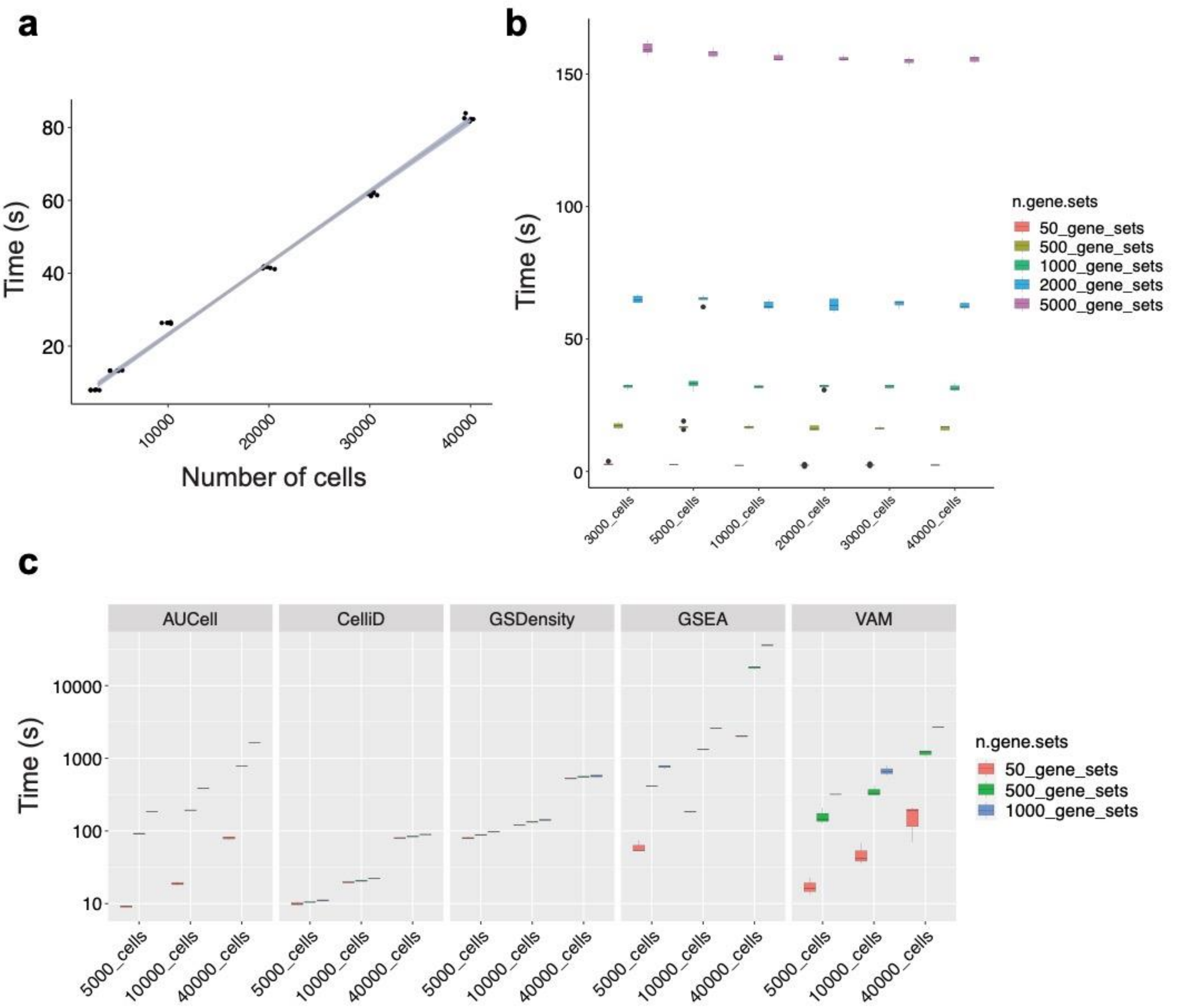

Supplementary Figure 23. Runtime for GSDensity and other pathway analysis methods. Source data are provided as a Source Data file.

a. Runtime for MCA embedding calculation with different number of cells as the input.

b. Runtime for gene set coordination test with different number of cells and gene sets as the input. n = 3 for each box.

c. Runtime for pathway activity scoring in five methods. For visualization purpose, the y-axis was log-transformed. n = 3 for each box.

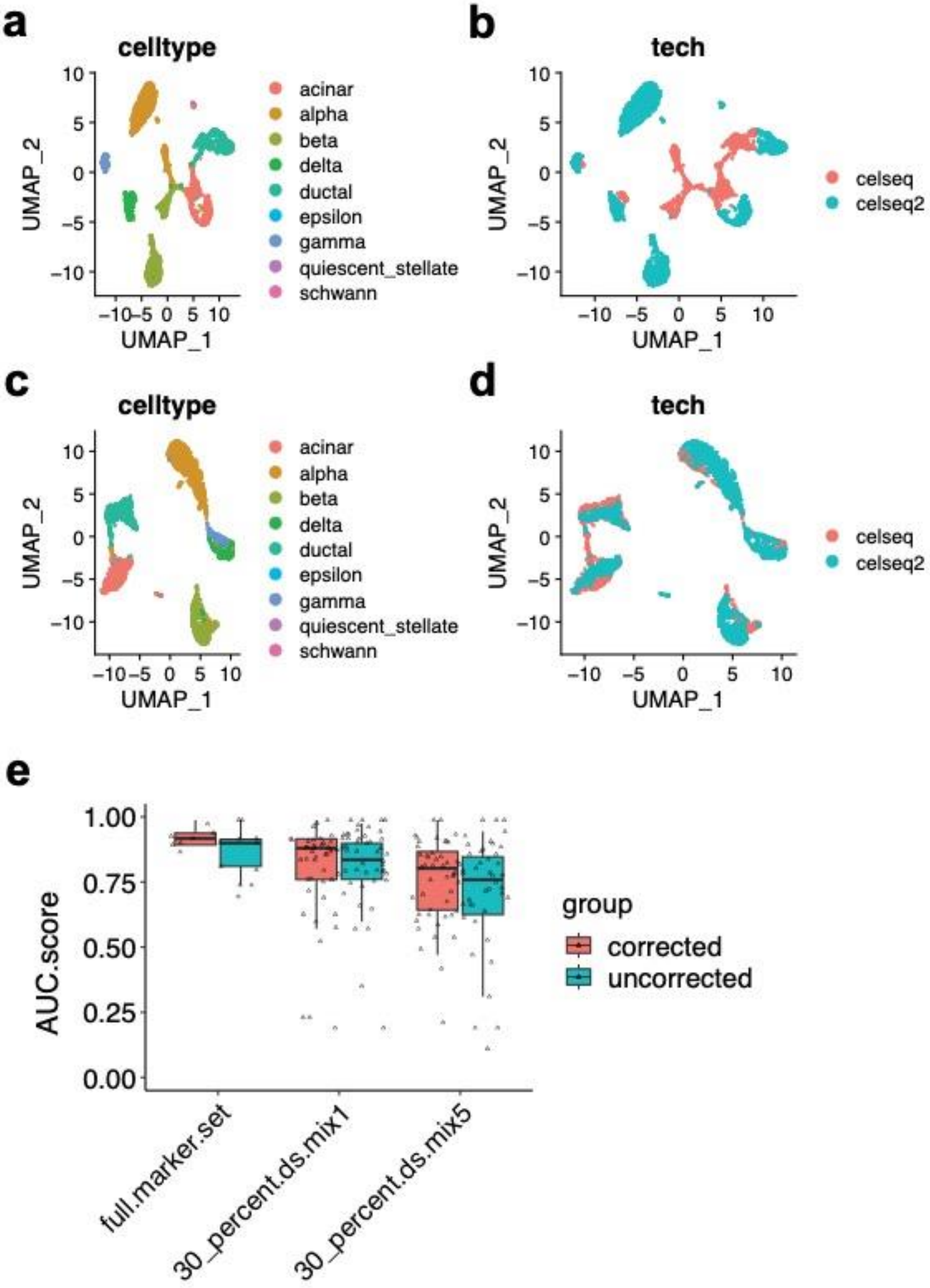

Supplementary Figure 24. Performance of GSDensity in data with batch effect before and after batch correction.

a-b. UMAP demonstration of a human pancreas dataset (uncorrected). The cells were colored by annotated cell types (a) or sequencing technology (b).

c-d. UMAP demonstration of a human pancreas dataset (corrected). The cells were colored by annotated cell types (c) or sequencing technology (d).
e. The cell identity recovery using marker genes (AUC metric) in batch-corrected and uncorrected data. We used full marker sets and marker sets mixing with 1:1 and 1:5 random genes. Source data are provided as a Source Data file.

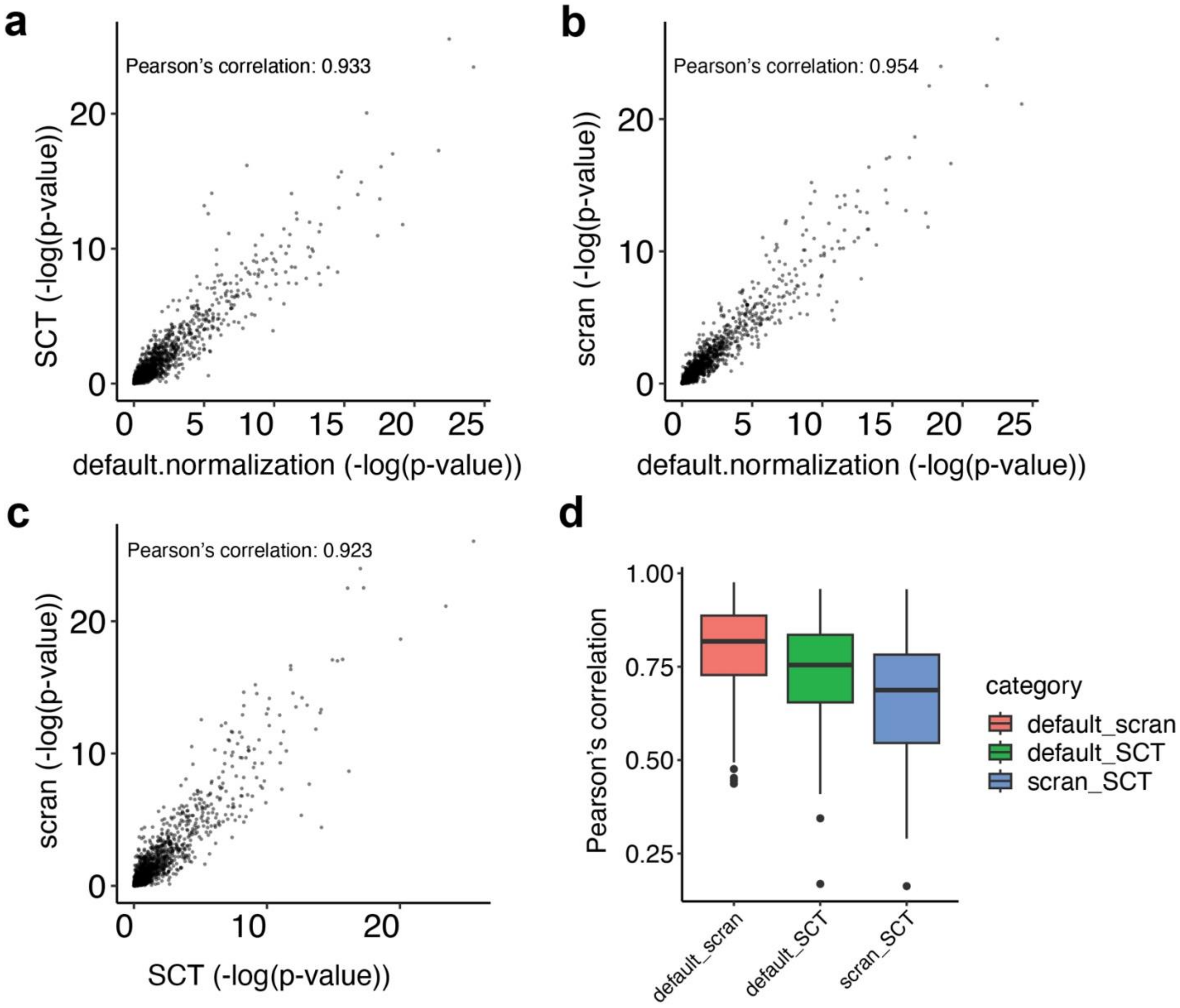

Supplementary Figure 25. Performance of GSDensity using different normalization strategies for scRNA-seq data.
a-c. Correlation of gene set coordination calculation between default Seurat ('default') and SCTransform ('SCT') (**a**), default and scran (**b**), and SCT and scran (**c**). We used the PBMC3K data. Negative log-transformed p-values were used as the metric of gene set coordination. The first 2,000 GO Biological Processes gene sets were used, with 1,751 passing the filter of at least 3 genes from the list being detected.
d. Correlation of gene set pathway activity levels (PALs) at single cell levels. For the gene sets used in a-c, 224 showed strong coordination (p-value < 0.01, one-sided t-test). n = 224 for each box. PALs were calculated in the PBMC3K datasets with different normalization strategies. Pearson's correlation coefficients were calculated for each of the 224 gene sets comparing default and scran, default and SCT, and scran and SCT.

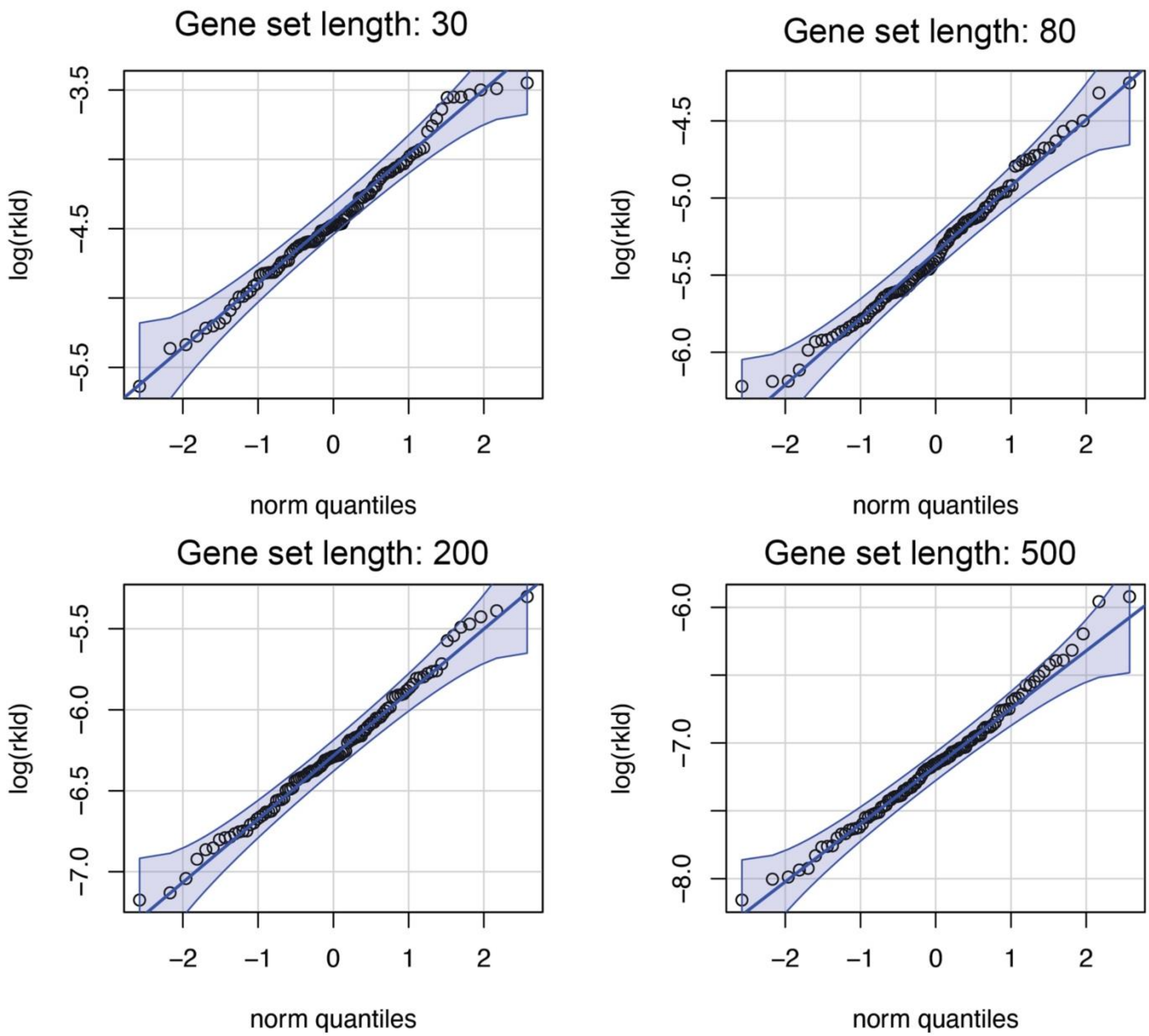

Supplementary Figure 26. The distribution of log-transformed KL-divergence $D_{KL}(P_r||Q)$ between the density of randomly sampled genes and background genes. The four panels showed the qqplot for random gene sets of different lengths, according to the length of most of the publicly curated gene sets.

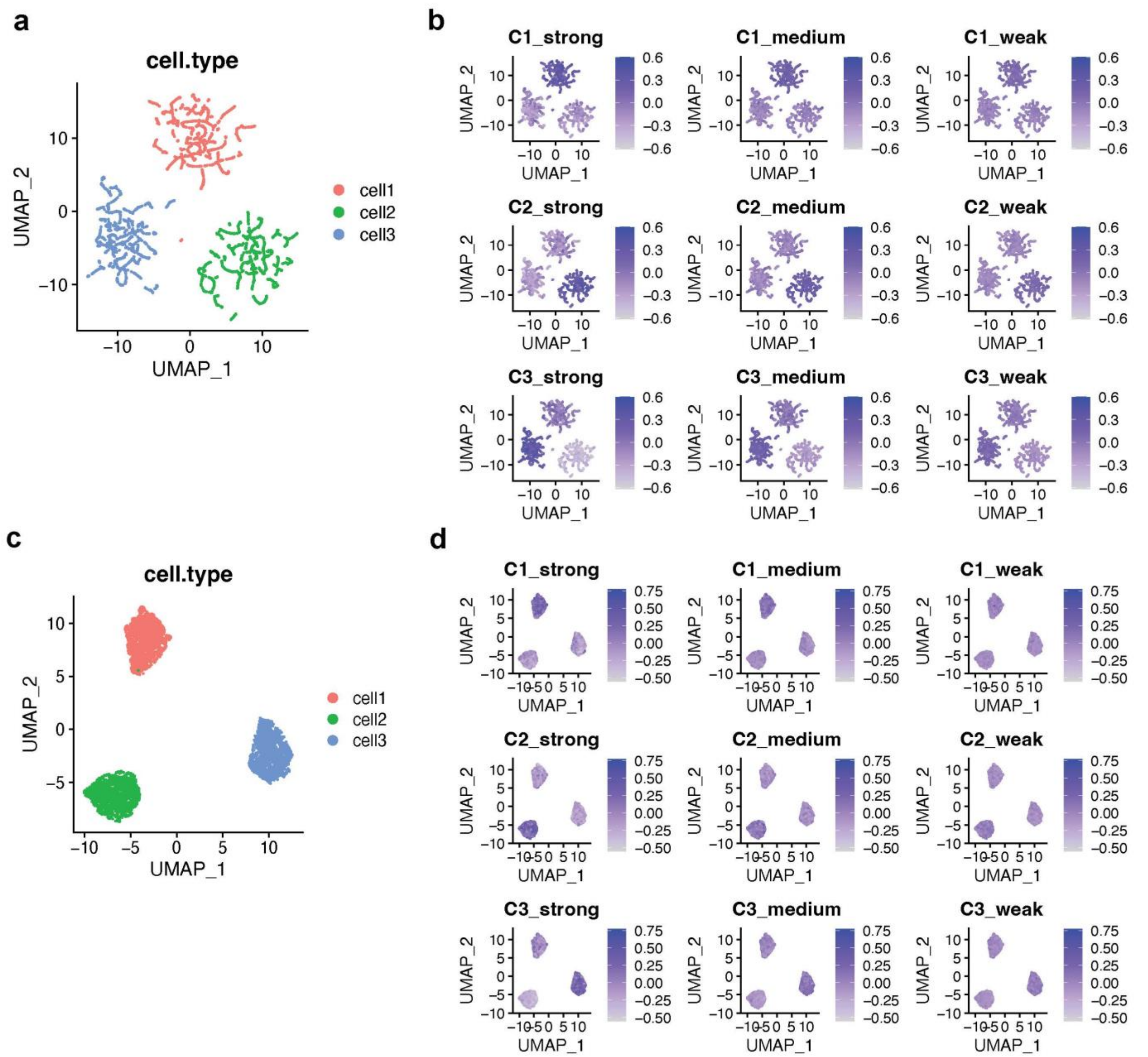

Supplementary Figure 27. Demonstration of simulated scRNA-seq data and marker sets for Mode-1: steady state scRNA-seq data.

a. UMAP visualization of simulated ‘clean’ dataset of Mode-1. Three such datasets were simulated and here we only showed the first batch. The UMAP embeddings were calculated based on gene expression.

b. The overall expression level of strong, medium, and weak marker sets for each cluster for the clean matrix. The expression levels were calculated using the ‘AddModuleScore’ function in Seurat.

c. UMAP visualization of the simulated Mode-1 dataset with noise added. We added noises of different levels to the same clean matrix and resulted in different drop-out rates of the final matrix. Here we only showed the result with 65% drop-outs. The UMAP embeddings were calculated based on gene expression.

d. The overall expression level of strong, medium, and weak marker sets for each cluster for the noise-added matrix. The expression levels were calculated using the ‘AddModuleScore’ function in Seurat.

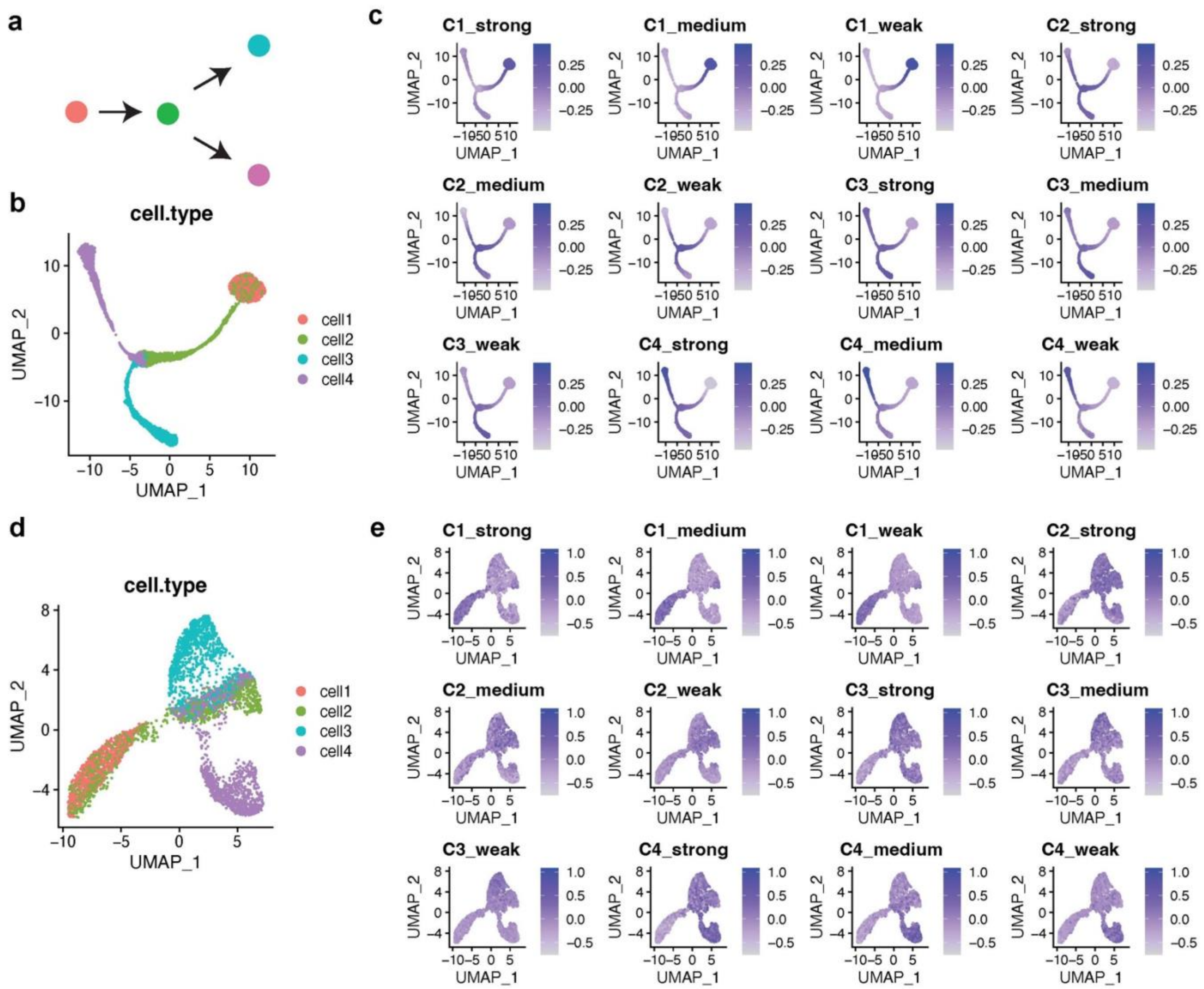

Supplementary Figure 28. Demonstration of simulated scRNA-seq data and marker sets for Mode-2: dynamic scRNA-seq data with four cell states in a bifurcation model.

a. Schematic of the bifurcation model.

b. UMAP visualization of simulated ‘clean’ dataset of Mode-2. Three such datasets were simulated and here we only showed the first batch. The UMAP embeddings were calculated based on gene expression.

c. The overall expression level of strong, medium, and weak marker sets for each cluster for the clean matrix. The expression levels were calculated using the ‘AddModuleScore’ function in Seurat.

d. UMAP visualization of the simulated Mode-2 dataset with noise added. We added noises of different levels to the same clean matrix and resulted in different drop-out rates of the final matrix. Here we only showed the result with 65% drop-outs. The UMAP embeddings were calculated based on gene expression.

e. The overall expression level of strong, medium, and weak marker sets for each cluster for the noise-added matrix. The expression levels were calculated using the ‘AddModuleScore’ function in Seurat.

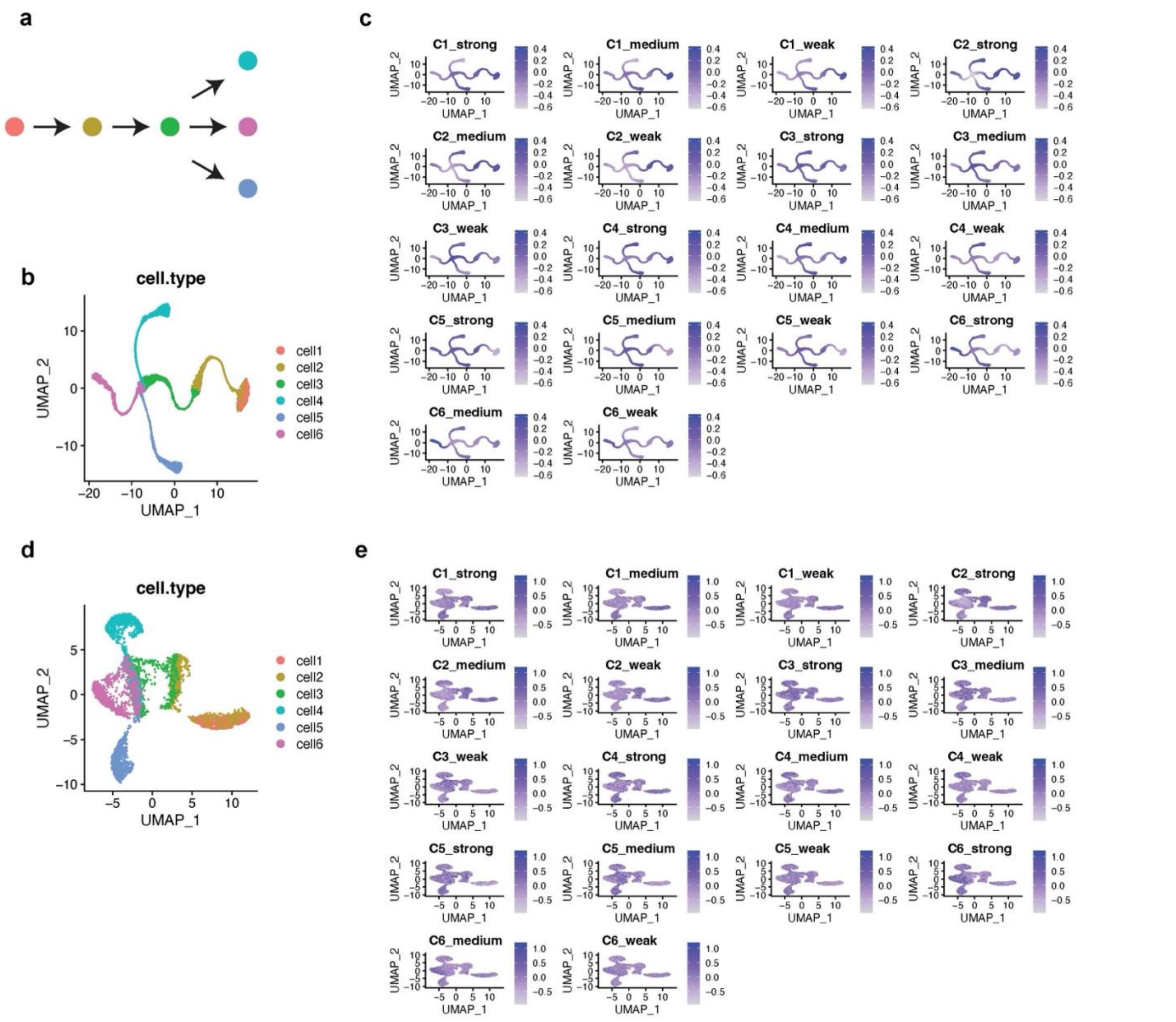

Supplementary Figure 29. Demonstration of simulated scRNA-seq data and marker sets for Mode-3: dynamic scRNA-seq data with six cell states in a trifurcation model.

a. Schematic of the bifurcation model.

b. UMAP visualization of simulated ‘clean’ dataset of Mode-3. Three such datasets were simulated and here we only showed the first batch. The UMAP embeddings were calculated based on gene expression.

c. The overall expression level of strong, medium, and weak marker sets for each cluster for the clean matrix. The expression levels were calculated using the ‘AddModuleScore’ function in Seurat.

d. UMAP visualization of the simulated Mode-3 dataset with noise added. We added noises of different levels to the same clean matrix and resulted in different drop-out rates of the final matrix. Here we only showed the result with 65% drop-outs. The UMAP embeddings were calculated based on gene expression.

e. The overall expression level of strong, medium, and weak marker sets for each cluster for the noise-added matrix. The expression levels were calculated using the ‘AddModuleScore’ function in Seurat.
